# Exploiting the drought tolerance of wild *Elymus species* for bread wheat improvement

**DOI:** 10.3389/fpls.2022.982844

**Published:** 2022-10-06

**Authors:** Ajab Khan, Ahmad Ali, Zahid Ullah, Iftikhar Ali, Prashant Kaushik, Mohammed Nasser Alyemeni, Awais Rasheed, Hassan Sher

**Affiliations:** ^1^Center for Plant Sciences and Biodiversity, University of Swat, Swat, Pakistan; ^2^State Key Laboratory of Molecular Developmental Biology, Institute of Genetics and Developmental Biology, Chinese Academy of Sciences, Beijing, China; ^3^Instituto de Conservación y Mejora de la Agrodiversidad Valenciana, Universitat Politécnica de Valéncia, Valencia, Spain; ^4^Botany and Microbiology Department, King Saud University, Riyadh, Saudi Arabia; ^5^Department of Plant Sciences, Quaid-i-Azam University, Islamabad, Pakistan

**Keywords:** crop wild resources, *Elymus* species, bread wheat, drought stress, leaf and culm anatomy

## Abstract

Crop wild resources are excellent sources of new genetic variation for resilience against climate extremes. However, detailed characterization of the desirable phenotypes is essential before using these crop wild resources in breeding programs. This current study was, therefore, conducted to investigate the water stress responses of eight wild *Elymus* species and two wheat cultivars. The experiment was carried out under varying levels of osmotic stress induced by polyethylene glycol and progressive water stress through different field capacities. Water stress significantly reduced both physiological and biochemical traits compared to control, ranging from 7.1% (protein content) to 34.5% (chlorophyll) under moderate stress and 9.1–45.8% under severe stress. The anatomical features were also affected under progressive water stress, including a reduction in xylem vessel diameter (7.92 and 16.50%), phloem length (4.36 and 7.18%), vascular bundle length (3.09 and 6.04%), and ground tissue thickness (2.36 and 5.52%), respectively. Conclusively, *Elymus borianus* (endemic to Swat, Pakistan)*, E*. *russelli, E*. *caninus, E*. *longioristatus*, and *E*. *dauhuricus* outperformed the check wheat cultivar, Pirsabak 2005, which is a rainfed variety. The results revealed that *Elymus* species belonging to the tertiary gene pool of bread wheat could be an excellent drought tolerance source for use in a breeding program.

## Introduction

Wheat (*Triticum aestivum* L.) is the most widely grown cereal crop and essential staple food, with a global production of more than 768 million metric tons per annum (Erenstein et al., 2022). The wheat requirement increases with each passing day due to stagnant yield per unit area and a rise in the global population (Ray et al., 2015). It has been estimated that worldwide wheat production needs to be increased by 60% to feed a population of 9.1 billion by the year 2050 (Grote et al., 2021). Drought stress affects morphological and anatomical features of roots, stems, and leaves, which ultimately reduces plant physiological functions (Ali et al., 2013; Salam et al., 2022). Drought stress at pre-anthesis reduces grain yield by affecting photosynthetic activity, and decreasing the accumulation of reserves in the stem (Ehdaie et al., 2006; Ali et al., 2014a; Fatima et al., 2014). At reproductive stages, the wheat crop is highly vulnerable to drought stress, which ultimately reduces the fertility of spikelet, the weight of a single grain, and the duration of grain filling (Prasad et al., 2011; Mahrookashani et al., 2017). Further, drought stress also affects flowering times in plants (Ali et al., 2022a,b). It increases the production of harmful substances like reactive oxygen species (ROS), which damages the metabolic activities, structures, and functions of plant cells (Suneja et al., 2017). Plants produce different types of antioxidants to detoxify ROS and protect themselves from its negative effects by inducing drought tolerance (Ashraf, 2010; Verma et al., 2014). Under drought stress, increased accumulation of antioxidants results in ROS scavenging, which may lead to increased proline and decreased protein content in plants (Hessini et al., 2009; Zhang and Nan, 2010).

Establishing the agronomic superiority of wheat germplasm derived from crop-wild introgression under abiotic stress conditions is of prime importance in breeding (Afzal et al., 2018). Utilization of wild crop resources is a promising approach for producing improved crop varieties (Masoomi-Aladizgeh et al., 2015). Crop wild resources can provide a variety of traits to cultivated crops with the potential to decrease yield loss due to various environmental stresses (Kiani et al., 2015; Rafique et al., 2017; Mammadov et al., 2018). Genetic diversity in wild relatives of wheat is an ideal source for producing new tolerant varieties predominantly for drought, salinity, cold, and heat stresses (Rasheed et al., 2018). Wheat wild relatives are categorized into three gene pools: primary, secondary, and tertiary gene pools (Zhang et al., 2017). The primary gene pool includes hexaploid landraces, cultivated tetraploids, A and D genome diploid donors, and wild *T. dicoccoides*. The secondary gene pool includes *Aegilops* species and polyploid *Triticum*. Further, the tertiary gene pool includes *Secale cereale, Thinopyrum intermedium*, and species of the genus *Elymus* (Chaudhary et al., 2014).

*Elymus L*. is the largest genus of the family *Poaceae* and tribe *Triticeae*, comprising about 150 species mainly distributed in temperate zones of the northern hemisphere (Dewey, 1984). Cytologically, species of the genus *Elymus* contain St, H, Y, P, and W subgenomes from *Pseudoroegneria, Hordeum, Agropyron*, and *Australopyrum* genomes, respectively (Jensen, 1990). In Pakistan, the genus is represented by 22 species (Cope, 1982). Due to compact and determinate floral structure, self-pollination ability, and other characteristics, several *Elymus* species have been developed as forage cultivars, including *E. glaucus, E. lanceolatus, E. Canadensis, E. trachycaulus, E. wawawaiensis*, and *E. virginicus* (Aubry et al., 2005). Species of the genus *Elymus* have close taxonomic relations to wheat, rye, and barley and thus serve as a genetic reservoir of potential alien genes for improving these crops against stresses and other agronomic traits (Sasanuma et al., 2002). Recently, the introgression of *Elymus repens*-3St chromatin into wheat and its characterization by Gong et al. (2019) has demonstrated high resistance to stripe rust and *Fusarium* head blight by the translocation lines in comparison to the wheat parent.

Species of the genus *Elymus* have received much attention, as they carry various characteristics such as resistance to environmental stresses, diseases, and pests (Schooler and Anderson, 1980; Aung, 1991). Some species like *Elymus dahuricus, E. nutans*, and *E. elongatus* are considered potential sources for desirable traits like salinity, drought, and cold tolerance (Gorham et al., 1984; Gazanchian et al., 2007). Therefore, these species might be beneficial as tertiary genetic resources for improving common bread wheat and other forage grasses (Zhao et al., 2013). Drought adaptability of the *Elymus species* may be explored through detailed characterization of its physio biochemical and anatomical attributes is essential. The current study aimed to investigate the water stress responses of *Elymus* species and explore potential species to be utilized in wheat breeding for drought tolerance.

## Materials and methods

### Plant specimen and seed collection

Plant samples and seeds of *Elymus* (Triticeae) were collected from northern Pakistan's temperate and alpine regions in July–September 2019. Northern Pakistan encompasses northern Khyber Pakhtunkhwa and Gilgit-Baltistan. Geographically, the region lies in a temperate climate zone, between 34° and 37°N and 71.4° and 77.6°E, with high alpine meadows, glaciers, snow-covered peaks, high waterfalls, and coniferous forests (Ullah et al., 2015). The region is a meeting point for the world's three highest mountain ranges, i.e., the Himalayas, Karakorum, and Hindukush, and is home to some of the highest peaks, including K2, the world's 2nd highest peak. The region is also rich in diverse ethnicities, languages, cultures, and religious beliefs (Abbas et al., 2016). The huge topographic, edaphic, and climatic variability gives rise to diverse and rich flora and fauna. The climate in the upper elevations is extremely harsh, with little rain and plenty of snow, and the areas are described as snow deserts. The temperature in winter may fall well below freezing. The summers in low-lying areas are pleasant. These mountain ecosystems are home to every third species of plant and provide plenty of ecosystem services (Khan et al., 2012). *Elymus* is a common component of these alpine and subalpine meadows.

A sampling of plants and seeds was collected from eight *Elymus* species, including *Elymus longiaristatus* (Boiss.) Tzvelev, *E. dahuricus* Griseb., *E. schrenkianus* (Fisch. & C.A.Mey.) Tzvelev, *E. semicostatus* (Nees ex Steud.) Melderis, *E. nutans* Griseb., *E. russelli* (Melderis) Cope*, E. borianus* (Melderis) Cope, and *E. caninus* (L.) L.), growing in wild habitats in northern Pakistan. During field collection, the field notebook noted geographic coordinates, associated species, phenology, and habitat features for each specimen locality. The specimens were dried and preserved, and voucher specimens were submitted to the Swat University Herbarium (SWAT). Botanical taxa were identified by expert taxonomists using the flora of Pakistan (Cope, 1982). Seeds of two wheat varieties, Pirsabak-2005 and Faisalabad-2008, were executed at the National Agriculture Research Center (NARC), Islamabad, Pakistan (Table 1).

**Table 1:** Locality/source and voucher numbers of the studied *Elymus* species and wheat cultivars.

**S. No**.	**Voucher no**.	***Elymus species*/wheat cultivar name**	**Locality/source**
1	SWAT007001	*E. longiarsitatus* (Boiss.) Tzvelev	Utror valley Swat, Pakistan
2	SWAT007002	*E. dahuricus* Griseb.	Near Kachura Lake Skardu, Gilgit Baltistan, Pakistan
3	SWAT007003	*E. schrekianus* (Fisch. & C.A.Mey.) Tzvelev	Deosai plateau, Skardu, Gilgit Baltistan, Pakistan
4	SWAT007004	*E. semicostatus* (Nees ex Steud.) Melderis	Mindam valley Swat, Pakistan
5	SWAT007005	*E. nutans* Griseb.	Utror valley Swat, Pakistan
6	SWAT007006	*E. russelli* (Melderis) Cope	Utror valley Swat, Pakistan
7	SWAT007007	*E. borianus* (Melderis) Cope	Utror on way to Kandol lake Swat, Pakistan
8	SWAT007008	*E. caninus* (L.) L.	Mankyal valley Swat, Pakistan
9		Pirsabak-2005	PGRI, NARC Islamabad, Pakistan
10		Faisalabad-2008	PGRI, NARC Islamabad, Pakistan

### Germination experiment

Seeds of eight *Elymus* species and two wheat genotypes were sterilized for 10 min with 5% sodium hypochlorite (NaOCl) and rinsed thoroughly with deionized water. Ten seeds of each species replicated three times were germinated under osmotic stress conditions induced by polyethylene glycol (PEG_6000_). Three treatments, i.e., 0% (control), 7.5%, and 15% PEG (w/v), were used in a completely randomized design (CRD) to explore variations in germination characteristics of the experimental plant (Ali et al., 2015). Germination percentage, GP (Larsen and Andreasen, 2004); mean germination time, MGT (Ellis and Roberts, 1981); and germination index, GI [Association of Official Seed Analysts (AOSA), 1983] were calculated accordingly.

### Pot experiment

An experiment was carried out in a greenhouse at the Center for Plant Sciences and Biodiversity, the University of Swat, to compare the physio, biochemical, and anatomical responses of the experimental plant materials, comprising *Elymus* species and wheat cultivars, under water stress conditions. Experimental soil used as a growth medium in the experiment was collected from the Agriculture Research Institute Mingora Swat, Pakistan, which was thoroughly examined for its physical and chemical properties (Supplementary material 1). Seeds were germinated for all the species, and uniform-sized seedlings were transplanted into plastic pots (24 × 18, 30 cm) filled with the same soil and exposed to sunlight for better growth with an average daily temperature of 20/26°C and 65% humidity. The field capacity (FC) of the soil was determined gravimetrically, as previously discussed in Graber et al. (2010). Treatments in the pot experiment were applied as previously reported in Liu and Li (2005), which included control (80% FC, i.e., well-watered condition), moderate stress (MS; 55% FC), and severe stress (SS; 30% FC). The experimental design employed during the study was a randomized complete block design (RCBD) replicated three times. Accordingly, each pot was watered to its field capacity until 60 d of seedlings were transferred, after which water stress was initiated. Water stress was monitored by weighing each pot daily and adding water lost by evapotranspiration till the respective FC regimes were retained. Normal weeding and agricultural practices were carried out to provide proper environmental conditions. Thirty days after the imposition of stress, when wilting and curling appeared in SS treatment, an indication of permanent wilting, leaf samples were collected, weighted, wrapped in aluminum foil, and kept in a refrigerator for determination of various anatomical, physiological, and biochemical attributes.

### Micromorphological characterization of leaf epidermis

For obtaining epidermal leaf peel, about a 2 cm long segment from the mid-portion of five mature leaves was taken and boiled in 88% lactic acid in a water bath at 100°C for about 30 min (Clark, 1960). The leaves, softened with lactic acid, were subjected to the removal of abaxial and adaxial epidermal peels using a sharp razor blade. Epidermal peels were stained with 1% safranin and placed in glycerol or lactic acid on glass slides. Qualitative and quantitative features of the epidermis were observed and measured using an Olympus light microscope equipped with a digital camera (Meiji infinity DK-5000, Japan). Qualitative traits included cell shape, cell wall morphology, presence, and absence of stomatal complexes and prickles, while quantitative traits included long cell length (LCL), long cell width (LCW), stomatal complex length (SCL), and stomatal complex width (SCW).

### Anatomical characterization of leaves and culm

An anatomical investigation of leaves and culm from all experimental plant materials was carried out to explore variations in response to drought stress treatments following the method of Ruzin (1999). This was accomplished by taking small fragments of flag leaf and culm of all the studied plant species when permanent wilting appeared under severe stress treatment. Specimens were then fixed in formalin acetic alcohol (FAA) solution, which comprised formalin (10 mL), 70% ethyl alcohol (85 mL), and glacial acetic acid (5 mL) for 24 h. Freehand sectioning was employed for cutting sections (~20 μm), which were then serially dehydrated by ethanol (30, 50, 70, and 90%, respectively). In order to enhance visibility, the sections were double stained with safranin—light green. The prepared slides were then analyzed through a light microscope, which was followed by photomicrography for five sections per replication. Various internal anatomical features of the culm, including phloem length (PL), phloem width (PW), vascular bundle length (VBL), vascular bundle width (VBW), and ground tissue thickness (GT), were recorded. Similarly, leaf upper epidermis thickness (UET), lower epidermis thickness (ET), xylem vessels diameter (XVD), phloem length (PL), phloem width (PW), vascular bundle length (VBL), vascular bundle width (VBW), and mesophyll thickness (MT) were also recorded.

### Physiological and biochemical analysis

Chlorophyll content was measured by following the method described in Ali et al. (2014b) using dimethyl sulphoxide (DMSO). Proline content in leaf samples was determined using 3% sulfosalicylic acid according to the method of Bates et al. (1973). The determination of protein was performed following the method of Lowry et al. (1951), using bovine serum albumin (BSA) as a standard. Soluble sugar was determined by following the method described by Dubois et al. (1956) using phenol sulfuric acid. Antioxidant enzymes were extracted as described in Ali et al. (2014a,b), with minor modifications. ~0.5 g of fresh plant tissues were ground in 10 mL of 50 mM chilled phosphate buffer in an ice bath using a pestle and mortar. The homogenate was collected and filtered through cloth. It was then centrifuged at 15000 rpm for 25 min at 4 °C (Bian and Jiang, 2009). Finally, the supernatant was used for antioxidant enzymatic activity analysis. An assay for superoxide dismutase was carried out by using the method of Giannopolitis and Ries (1977). Peroxidase activity was determined by following the method described by Gorin and Heidema (1976).

### Statistical analysis

The obtained data were subjected to analysis of variance (ANOVA) using STATISTIX 10.0. as described in Steel et al. (1997). The data was also subjected to principal component analysis (PCA) using a multivariate option in PAST v. 2.12. A heatmap of Pearson's correlation was created to visualize the association between different variables. Summary statistics of the studied anatomical traits were graphically visualized through box-violin plots, which were prepared by utilizing the Jamovi software (version 1.8; The jamovi project, 2021) and package *ggplot2* (Wickham et al., 2018). The relative difference among treatment means of all the studied attributes was calculated as previously described in Ahmadi et al. (2018) by using the formula:


(1) $$\begin{array}{l} RD=\frac{\bar{X}ww-\bar{X}ws}{\bar{X}ww}\times 100, \end{array}$$

where $\bar{X}ww\text{\_}$and $\bar{X}ws\text{\_}$ are the means of a studied trait under controlled and water stress treatments.

## Results

Results revealed that both osmotic and water stresses (moderate, FC 30%, and severe FC 50%) significantly affected all the studied morpho-physiological and biochemical attributes in *Elymus* species. and wheat genotypes. The mean square results of the studied traits showed that MGT, GI, GP, PRC, Chla, Chlb, TChl, and SOD were highly different in osmotic and water stress treatments. However, no significant differences were observed regarding SS, PC, and POD. Similarly, PC, PRC, Chla, Chlb, and TChl were found to be significantly different in water stress treatments (Table 2).

**Table 2:** Analysis of variance (mean squares), mean values, and percentage change in biochemical and physiological traits in the studied *Elymus species* and wheat cultivars.

**Source of variation**	**DF**	**MGT**	**GI**	**GP**	**SS**	**PC**	**PRC**	**Chla**	**Chlb**	**Tchl**	**POD**	**SOD**
Plant materials (MS)	9	37.67***	12.87***	7028.2***	50.3^NS^	21.6^NS^	295.3**	2.05***	0.45*	3.491***	14474.4^NS^	34451.9***
Drought stress (MS)	2	6.62^NS^	0.29^NS^	441.6^NS^	89.9^NS^	58.1*	446.4*	5.03***	0.65*	9.33***	7578.5^NS^	9704.7^NS^
Drought stress x plant materials (MS)	18	1.65^NS^	2.00^NS^	319.6^NS^	12.3^NS^	5.2^NS^	81.9^NS^	0.34^NS^	0.45^NS^	0.39^NS^	1450.7^NS^	944.1^NS^
Error	58	6.65	1.79	226.4	41.7	14.4	100.8	0.50	0.188	0.72	8666.9	5683.9
Control (MV)		4.08	2.69	73.8	12.1	29.1	20.4	1.64	0.72	2.36	109.45	57.75
Moderate drought stress (MV)		4.22	2.85	74.78	10.2	27.0	24.2	1.07	0.50	1.58	123.07	82.46
Severe drought stress (MV)		4.95	2.87	67.7	8.7	26.4	28.2	0.84	0.43	1.28	141.14	92.75
%age change due to moderate stress		– 3.31	– 5.95	– 1.3	16.1	7.1	– 18.4	34.52	29.80	33.05	– 12.44	– 42.79
%age change due to severe stress		– 21.36	– 6.66	8.3	28.5	9.1	– 37.7	48.62	39.41	45.82	– 28.95	– 60.59

Where; ^*^, ^**^ and ^***^ depict statistical significance at the 0.05, 0.01 and 0.001 probability level; NS, non-significant; MS, mean squares; MV, mean values.

MGT, Mean germination time; GI, Germination index; GP, Germination percentage; SS, Soluble sugar; PC, Protein contents; PRC, Proline contents; Chla, Chlorophyll a; Chlb, Chlorophyll b; TChl, Total chlorophyll; POD, Peroxidase activity; SOD, Superoxide dismutase activity.

### Germination characteristics

Water stress increased mean germination time (MGT) and germination index (GI). However, the studied plant materials reduced germination percentage (GP) under osmotic stress conditions. The observed increase in MGT was 3.3%, GI 5.9% under moderate stress, while it was 21.4% and 6.7% under severe stress, respectively. However, mean GP declined by 1.3% in moderate and 8.3% under severe water stress (Table 2). A maximum increase in MGT (32.1%) was recorded in *E. schrekianus* under moderate stress, while a minimum increase of 4.4% was recorded in *E. borianus* (Table 3). Similarly, maximum increased GI (73.6%) was observed in *E. caninus*, while minimum increase (8.0%) in *E. borianus*. A maximum reduction in germination percentage of 5.6% was observed in *E*. *nutans* under moderate stress (7.5% PEG). Similarly, *E. semicostatus* exhibited a decline in germination percentage (31.2 and 81.2%) under moderate and severe osmotic stress, respectively (Table 3).

**Table 3:** Variability in mean germination time, germination index, germination percentage, chlorophyll contents, and antioxidants enzymatic activities among *Elymus species* and wheat cultivars under drought stress.

**Plant materials**	**MGT (d)**					**GI (d)**					**GP (%)**					**Chla (mg/g)**				
	**%age change to control**					**%age change to control**					**%age change to control**					**%age change to control**				
	**Control**	**MS**	**SS**	**MS**	**SS**	**Control**	**MS**	**SS**	**MS**	**SS**	**Control**	**MS**	**SS**	**MS**	**SS**	**Control**	**MS**	**SS**	**MS**	**SS**
*E. longioristatus*	3.56	5.10	5.53	30.28	35.71	2.53	3.73	3.70	32.14	31.53	70.00	73.33	70.00	– 4.76	0.00	1.37	0.85	0.87	37.89	36.73
*E. dahuricus*	4.15	4.95	4.37	16.13	5.00	3.23	3.80	3.90	14.91	17.09	76.67	76.67	90.00	0.00	– 17.39	1.27	1.00	0.88	20.87	30.83
*E. schrenkianus*	4.73	6.97	7.33	32.06	35.45	2.57	2.17	1.00	– 18.46	– 156.7	53.33	36.67	10.00	31.25	81.25	2.43	0.76	0.32	68.61	86.96
*E. semicostatus*	3.45	3.16	4.42	– 8.99	21.98	3.10	2.93	4.10	– 5.68	24.39	90.00	93.33	93.33	– 3.70	– 3.70	2.78	2.07	1.38	25.45	50.16
*E. nutans*	5.75	6.04	6.25	4.94	8.06	3.50	3.13	1.53	– 11.70	– 128.3	60.00	56.67	23.33	5.56	61.11	0.97	0.86	0.84	11.42	12.90
*E. russelli*	7.45	5.30	8.55	– 40.6	12.84	6.07	4.07	5.93	– 49.09	– 2.50	81.48	77.78	70.37	4.55	13.64	2.80	1.91	1.31	31.75	53.18
*E. borianus*	2.39	2.50	3.23	4.44	26.2	2.30	2.50	3.23	8.00	28.86	96.67	100.0	100.0	– 3.45	– 3.45	1.43	1.01	0.94	29.51	34.09
*E. caninus*	6.67	5.54	6.36	– 20.3	– 4.80	0.93	3.53	1.87	73.58	50.00	13.33	33.33	26.67	– 150.0	– 100.00	1.26	0.83	0.79	33.97	37.38
Pirsabak– 2005	1.27	1.43	1.90	11.63	33.33	1.27	1.43	1.90	11.63	33.33	100.00	100.0	100.0	0.00	0.00	1.22	0.79	0.63	35.29	47.99
Faisalabad– 2008	1.40	1.17	1.60	– 20.0	12.50	1.37	1.17	1.50	– 17.14	8.89	96.67	100.0	93.33	– 3.45	3.45	0.85	0.63	0.44	25.74	47.77
**Plant materials**	**Chlb (mg/g)**					**TChl (mg/g)**					**POD (units/g)**					**SOD (units/g)**				
	**%age change to control**					**%age change to control**					**%age change to control**					**%age change to control**				
	**Control**	**MS**		**MS**	**SS**	**Control**	**MS**	**SS**	**MS**	**SS**	**SS**	**MS**	**SS**	**MS**	**SS**	**Control**	**MS**	**SS**	**MS**	**SS**
*E. longioristatus*	1.03	0.79	0.73	22.87	28.83	2.40	1.64	1.60	31.47	33.35	80.95	90.41	112.1	– 11.68	– 38.41	48.91	58.87	67.46	16.90	27.50
*E. dahuricus*	0.57	0.57	0.65	0.55	– 14.7	1.84	1.57	1.53	14.57	16.73	72.44	81.98	94.94	– 13.18	– 31.07	33.68	43.91	31.17	23.28	– 8.05
*E. schrenkianus*	0.79	0.46	0.59	41.15	24.85	3.22	1.23	0.91	61.90	71.77	77.07	95.69	102.7	– 24.15	– 33.29	28.48	38.72	58.99	26.46	51.72
*E. semicostatus*	1.23	0.83	0.68	32.55	44.80	4.01	2.90	2.06	27.63	48.52	105.33	132.5	143.0	– 25.80	– 35.78	26.22	32.36	40.54	18.97	35.31
*E. nutans*	1.14	0.58	0.38	48.83	66.93	2.11	1.44	1.22	31.63	42.08	125.27	134.0	153.9	– 6.97	– 22.88	45.13	59.12	81.20	23.65	44.41
*E. russelli*	0.61	0.59	0.38	3.03	38.19	3.42	2.51	1.69	26.59	50.49	141.79	171.4	108.5	– 20.90	23.48	116.64	140.3	197.6	16.88	40.98
*E. borianus*	0.58	0.34	0.30	40.29	48.08	2.01	1.35	1.24	32.60	38.11	137.40	188.5	199.8	– 37.19	– 45.45	55.77	85.70	94.79	34.93	41.17
*E. caninus*	0.48	0.39	0.19	19.86	60.66	1.75	1.22	0.98	30.06	43.83	176.91	190.0	241.3	– 7.40	– 36.37	165.85	245.7	259.6	32.50	36.10
Pirsabak– 2005	0.44	0.27	0.25	38.44	43.88	1.66	1.06	0.88	36.13	46.89	64.17	61.05	144.4	4.85	– 125.04	27.23	54.63	56.23	50.16	51.57
Faisalabad– 2008	0.32	0.22	0.21	30.49	34.23	1.16	0.85	0.65	27.04	44.07	113.21	85.17	110.7	24.77	2.21	29.61	65.31	39.91	54.65	25.80

*Where*, MGT, Mean germination time; GI, Germination index; GP, Germination percentage; Chla, Chlorophyll a; Chlb, Chlorophyll b; Tchl, Total chlorophyll; POD, Peroxidase activity; SOD, Superoxide dismutase activity; MS, moderate water stress; SS, severe water stress.

### Photosynthetic pigments

Photosynthetic pigments were also affected by water stress in comparison to control treatment. The average Chla content declined by 34.5%, Chlb by 29.8%, and TChl by 33.1% under moderate stress, while the decrease was 48.6, 39.4, and 45.8% under severe stress, respectively (Table 2). The maximum reduction in Chla content (68.6 and 86.9%) among Elymus species was recorded in *E. schrenkianus* under moderate and severe water stress, respectively. However, *E. nutans* was the least affected species, exhibiting 11.4 and 12.9% reduced Chla. Similarly, a maximum percentage decrease in Chlb of 48.8 and 66.9% was also recorded in *E. nutans*. In *E. dahuricus*, Chlb was reduced by 0.5% only under moderate water stress; however, the reduction was 14.67% under severe water stress. A maximum percentage decrease in TChl was recorded in *E. schrenkianus*, reduced by 61.9 and 71.8% under moderate and severe stress, respectively (Table 3). SOD and POD activity increased by 42.8 and 12.4% in moderate and 60.6 and 28.9% under severe water stress, respectively. Maximum POD in terms of percentage change was 37.2 and 45.4%, reported in *E. borianus* under MS and SS, respectively. The maximum change in SOD activity was reported in *E. borianus* and *E. schrenkianus*, in which SOD activity increased by 34.9% under MS and 51.7% under severe water stress (Table 3).

### Biochemical attributes

Biochemical attributes were significantly affected by both moderate and severe water stresses. Protein contents declined by 7.1 and 9.1% in moderate and severe water stress, respectively (Table 2). A minimum reduction in PC (1.7 and 2.1%) was reported in *E. russelli* under MS and SS treatment, respectively, which was followed by *E. dauhuricus—*which exhibited a 2.0 reduced PC under MS treatment. Similarly, SS declined by 16.1 and 28.5% in moderate and severe water stress, respectively. A maximum decrease in SS (38.5 and 49.8%) due to moderate and severe water stress was observed in *E. semicostatus*. Proline content increased by 18.4% in moderate water stress and 37.7% in severe water stress. When a comparison was made with the control treatment, increased PRC (71.0, 54.9, 51.3, and 35.6%) was observed in *E. borianus, E. dahuricus, E. schrenkianus*, and *E. caninus* under MS and SS, respectively (Table 4).

**Table 4:** Variability in soluble sugars, protein content, and proline content in *Elymus species* and wheat cultivars under drought stress.

	**SS (Mg/gm)**					**PC (Mg/gm)**					**PRC (**μ**mol/g)**				
	**%age change to control**					**%age change to control**					**%age change to control**				
**Plant materials**	**Control**	**MS**	**SS**	**MS**	**SS**	**Control**	**MS**	**SS**	**MS**	**SS**	**Control**	**MS**	**SS**	**MS**	**SS**
*E. longioristatus*	9.04	6.79	5.88	24.90	34.91	28.51	26.71	25.89	6.29	9.20	16.55	22.11	26.98	– 33.57	– 63.02
*E. dahuricus*	12.57	8.15	6.95	35.18	44.74	25.78	25.27	24.04	1.97	6.75	14.82	22.97	26.67	– 54.95	– 79.91
*E. schrenkianus*	14.44	12.11	11.47	16.12	20.56	28.88	27.13	26.07	6.04	9.73	17.38	26.31	44.58	– 51.34	– 156.45
*E. semicostatus*	8.80	5.41	4.42	38.49	49.78	27.61	26.84	25.71	2.80	6.90	15.16	15.53	14.12	– 2.44	6.86
*E. nutans*	13.25	11.35	9.26	14.33	30.07	34.18	27.91	28.26	18.35	17.33	31.09	34.74	35.37	– 11.74	– 13.77
*E. russelli*	11.59	11.37	15.16	1.86	– 30.78	27.67	27.21	27.10	1.67	2.09	22.95	30.78	28.79	– 34.12	– 25.46
*E. borianus*	11.54	9.18	7.83	20.45	32.15	33.98	27.48	26.92	19.11	20.76	16.70	28.57	23.06	– 71.03	– 38.05
*E. caninus*	17.28	11.50	11.40	33.44	34.04	29f.37	28.57	27.67	2.72	5.80	32.19	20.91	36.94	35.03	– 14.73
Pirsabak– 2005	9.64	14.55	8.45	– 51.03	12.26	28.20	27.80	27.45	1.42	2.66	16.79	17.08	22.78	– 1.71	– 35.65
Faisalabad– 2008	13.06	11.24	5.84	13.91	55.26	26.74	25.56	25.22	4.41	5.65	20.99	23.37	22.50	– 11.29	– 7.17

Where SS, Soluble sugar; PC, Protein contents; PRC, Proline contents.

### Characterization of anatomical features

**Figure 1:**
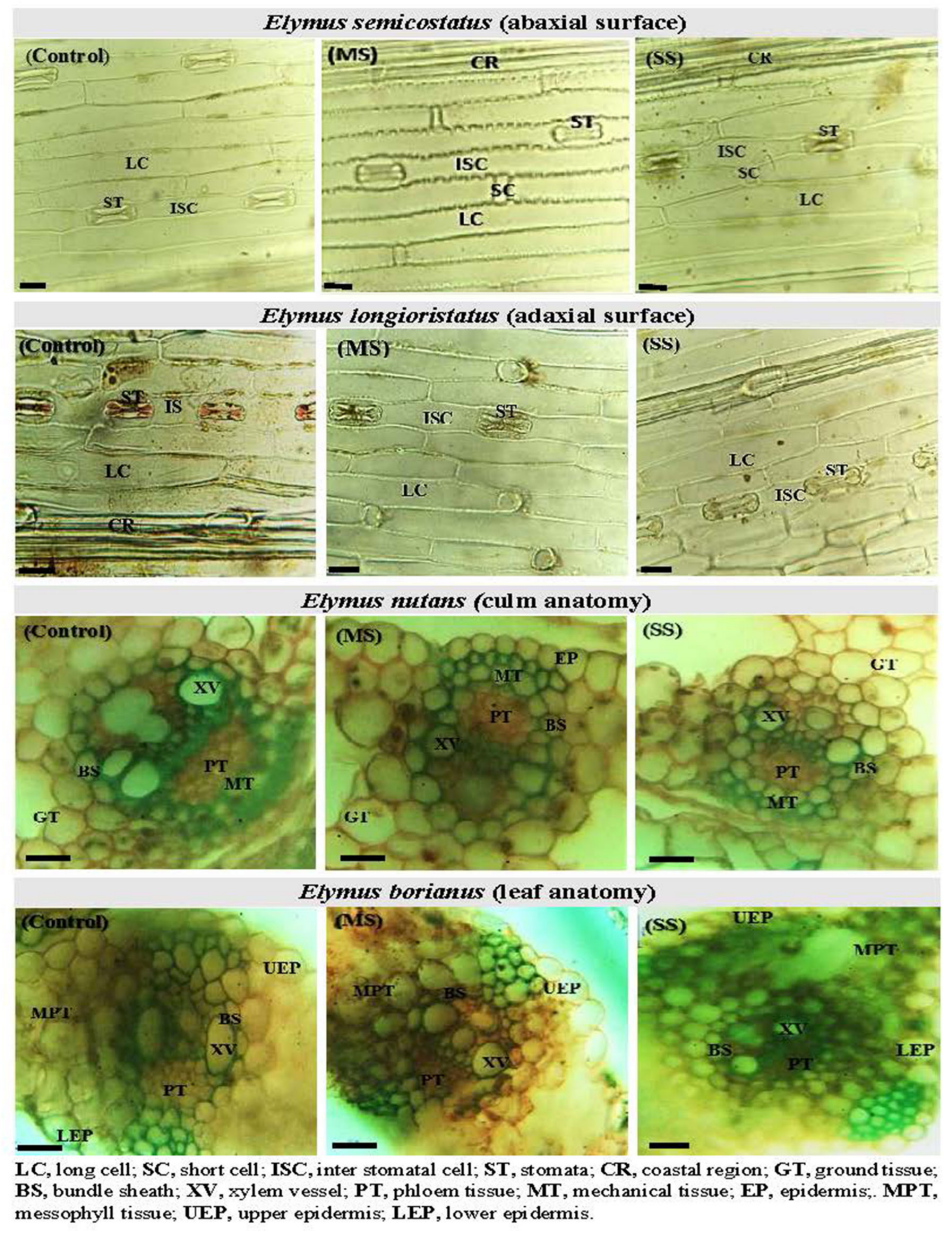
Leaf epidermal anatomy (abaxial surface and adaxial surface) of *Elymus longioristatus*; culm and leaf anatomy of *E. nutans* under control (C), moderate (MS), and severe (SS) water stress conditions. Bar scale 100 μm.

**Figure 2:**
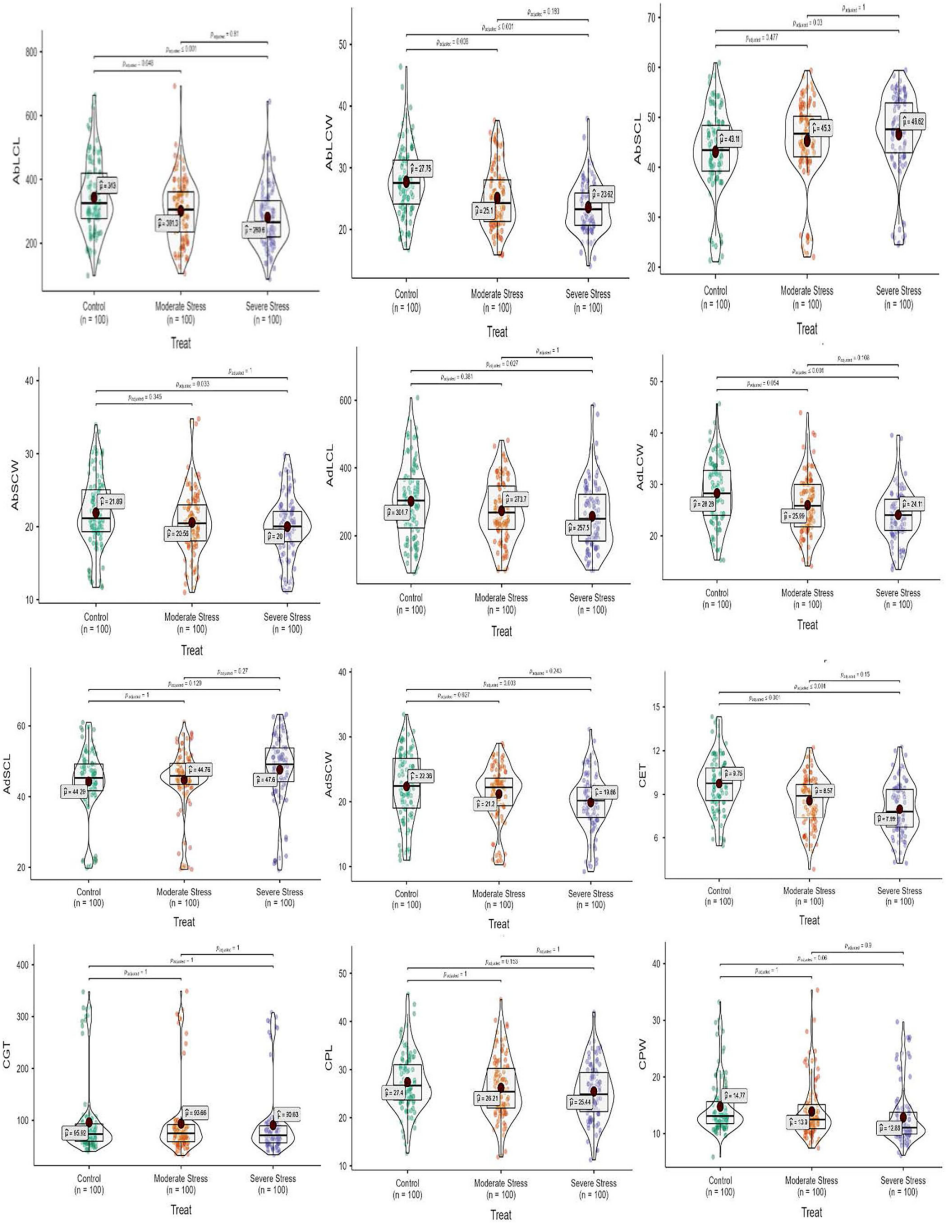
Box-violin plots presenting leaf epidermal (abaxial and adaxial surface) and culm anatomical features under control (C), moderate (MS), and severe (SS) water stress conditions. The interquartile range is depicted by the length of the boxes, the median by the horizontal line within the box, the mean by the red diamonds within the box, and the probability density of the data at different values by the widths of the violin plots.

Regarding leaf epidermal anatomy, high differences were revealed between experimental plant materials and drought stress treatments for the studied traits, including AbLCL, AbLCWD, AbSCL, AbSCW, AdLCL, AdLCW, AdSCL, and AdSCW. However, the interaction between *Elymus* species and drought stress treatments yielded differences with respect to AbSCL and AdSCL only. Among the studied traits, AbSCL and AdSCL were the most persistent, with an an18.8 and 20.9% coefficient of variation (CV), respectively. The long cell length of both abaxial and adaxial surfaces decreased by 12.2 and 9.3% in MS, while it was reduced by 18.2 and 14.6% in SS, respectively. Similarly, the LCW of both surfaces decreased by 9.6 and 8.1% in MS, while 14.9 and 14.8% in SS, respectively. The stomatal length of the abaxial and adaxial surfaces increased by 4.8 and 1.1% in MS, while 8.1% and 7.5% in SS, respectively. Stomatal width of the abaxial and adaxial surface declined by 6.1 and 5.2% in the MS, while it decreased by 8.6 and 11.1% in SS). In general, drought stress reduced all the traits of both epidermal surfaces in studied *Elymus* species and wheat genotypes (Figures 1, 2; Supplementary materials 2, 5).

**Figure 3:**
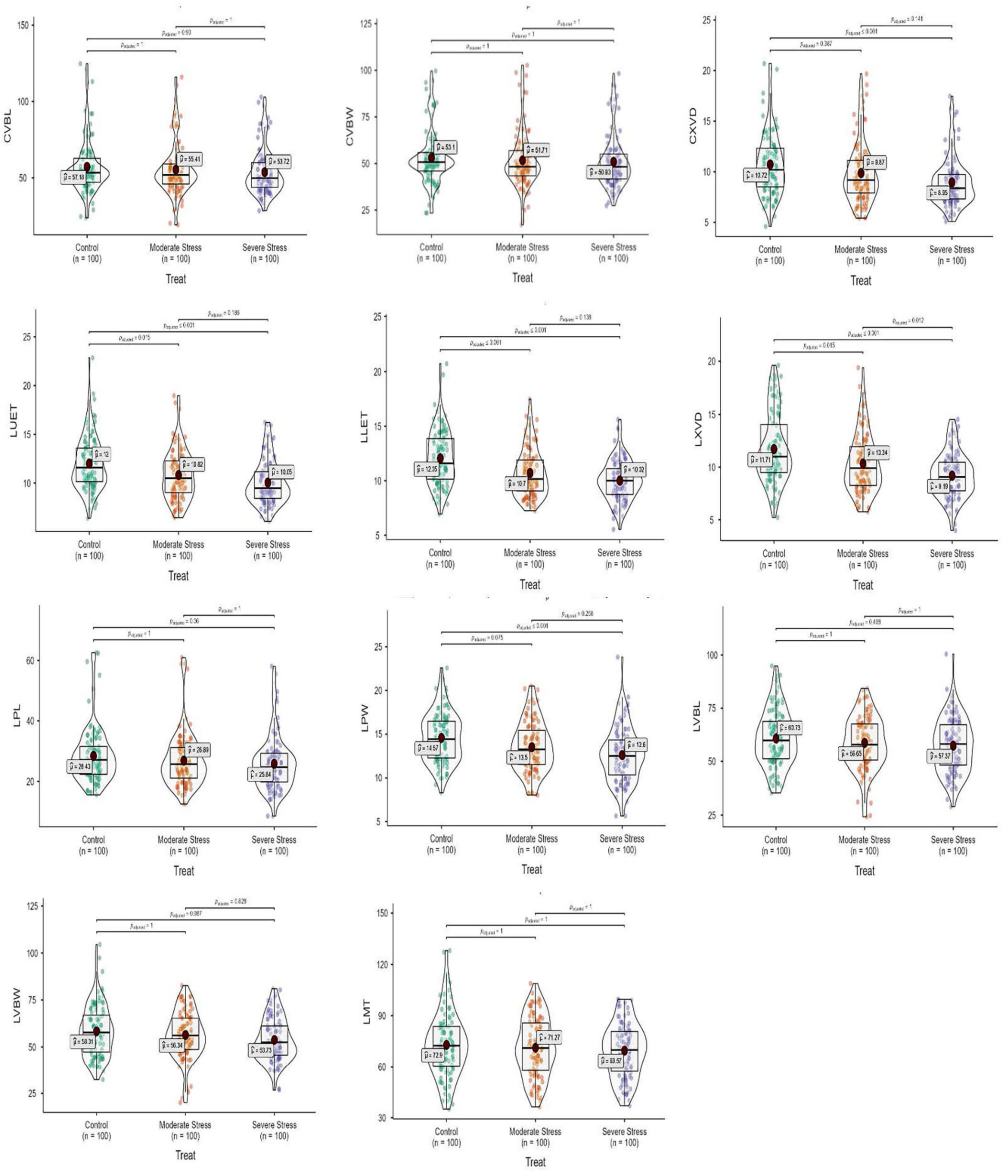
Box-violin plots presenting culm and leaf anatomical features under control (C), moderate (MS), and severe (SS) water stress conditions. The interquartile range is depicted by the length of the boxes, the median by the horizontal line within the box, the mean by the red diamonds within the box, and the probability density of the data at different values by the widths of the violin plots.

Mean squares of the culm anatomical features revealed that epidermal thickness, xylem vessel diameter, phloem length and width, vascular bundle length and width, and ground tissue thickness were highly different in the studied plant materials and drought stress treatments. However, no such difference was observed in the interaction between plants and stress treatments. The epidermal thickness of the culm declined by 12.1 and 18.0% under MS and SS, respectively. Culm xylem vessel diameter decreased by 7.9 and 16.5% in the MS and SS. Culm phloem length and width declined by 4.4 and 5.9% in MS and by 7.2 and 12.9 under SS, respectively. Similarly, vascular bundle length and width declined by 3.1 and 2.6% in MS, while 6.0 and 4.1% in SS, respectively. In general, drought stress reduced all culm anatomical traits in studied *Elymus* species and wheat genotypes (Figures 1–3; Supplementary materials 3, 6).

Mean squares of leaf anatomical traits indicated that lower and upper epidermal thicknesses, xylem vessel diameter, phloem length and width, vascular bundle length and width, and mesophyll tissue thickness were different among *Elymus* species and drought stress treatments. However, the interaction between the studied plant materials and drought stress treatments exhibited differences with respect to leaf xylem vessel diameter only. Leaf upper and lower epidermal thickness decreased by 9.8 and 11.1% in MS, while 16.2 and 16.8% in SS, respectively. Similarly, xylem vessel diameter declined by 11.7%, phloem length by 5.4%, phloem width by 7.3%, vascular bundle length by 3.4%, vascular bundle width by 3.4%, and mesophyll tissue thickness by 2.2% under MS, while these declined by 21.6, 9.1, 13.6, 5.5, 7.8, and 4.6% under SS, respectively. Generally, all the studied leaf vascular anatomical traits were significantly reduced under both water stress conditions (Figures 1, 3; Supplementary materials 4, 7).

### Correlation and principal component analysis

**Figure 4:**
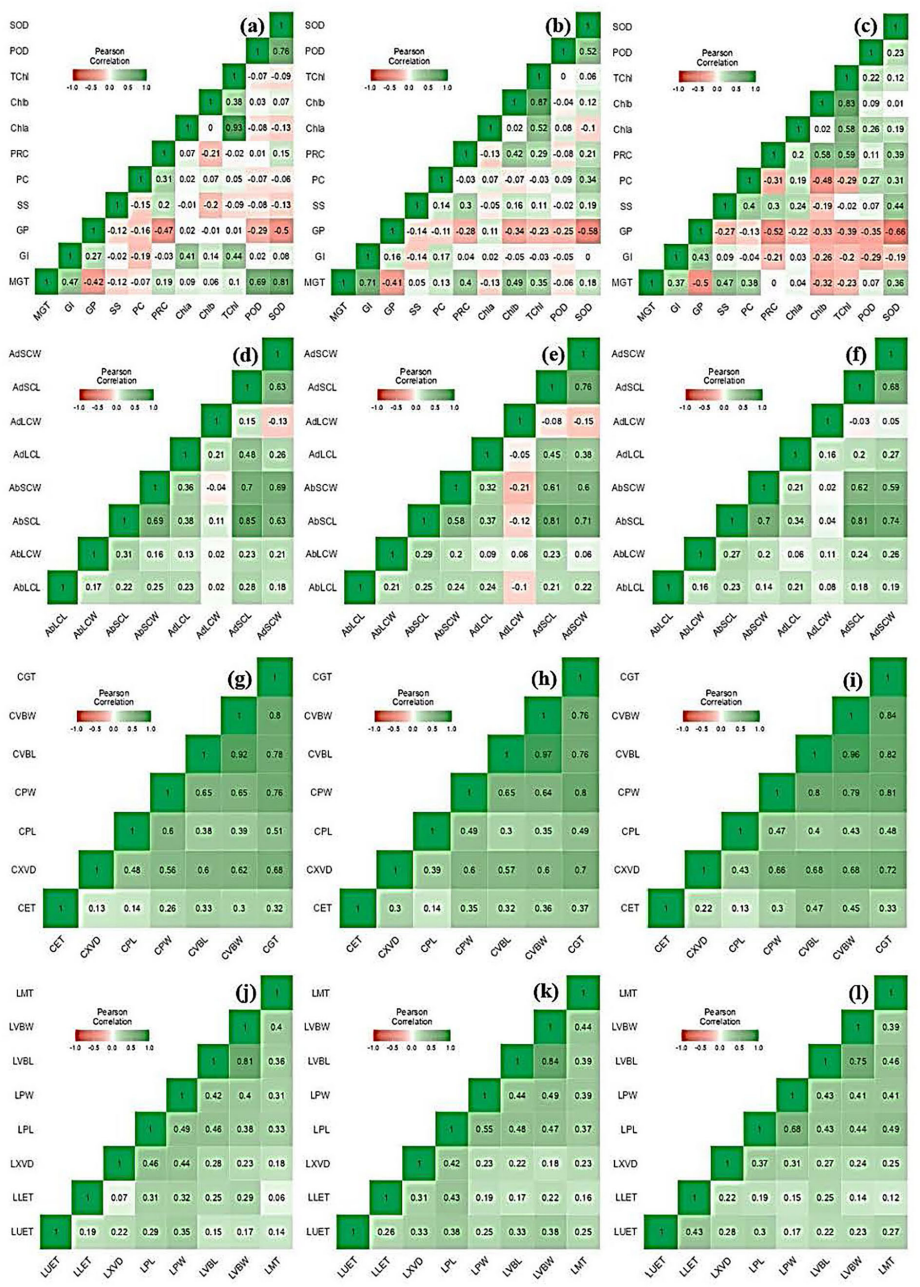
Heatmap showing Pearson correlation coefficient and associated probabilities among *Elymus species* and wheat cultivars for the studied traits using (*n* = 10) evaluated under osmotic (germination) moderate (MS; FC = 50%) and severe (SS; FC = 30%) water stress. **(a)** control, **(b)** MS, and **(c)** SS showing a relationship for morphological and physio biochemical traits; **(d)** control, **(e)** MS, and **(f)** SS showing a relationship for leaf micromorphological traits; **(g)** control, **(h)** MS, and **(i)** SS showing relationship for culm anatomical traits; and **(j)** control, **(k)** MS, and **(l)** SS showing relationship for anatomical and traits. The values in the color bar correspond to the Pearson correlation coefficient. Significant correlations are shown in green (positive) and red (negative) colors at *p* ≤ 0.05, ≤ 0.01, ≤ 0.001, respectively.

A heatmap showing the Pearson correlation coefficient and associated probabilities (*p* ≤ 0.05, ≤ 0.01, ≤ 0.001, respectively) among *Elymus* species and wheat cultivars for the studied traits is shown in Figure 4. The Pearson correlation showed the highest positive correlation between MGT and SOD (*r* = 0.812), followed by POD (*r* = 0.693) in the control condition, while it was non-significant under moderate and severe water stress. Similarly, a significant relationship was observed between MGT and GI (*r* = 0.707) in moderate water stress, which decreased in control (*r* = 0.473) and severe stress (*r* = 0.365), respectively. The relationship between POD and SOD (*r* = 0.759) was prominent in the control condition during its decline in moderate stress. A similar trend was also observed for anatomical traits, including CXVD and LXVD.

**Figure 5:**
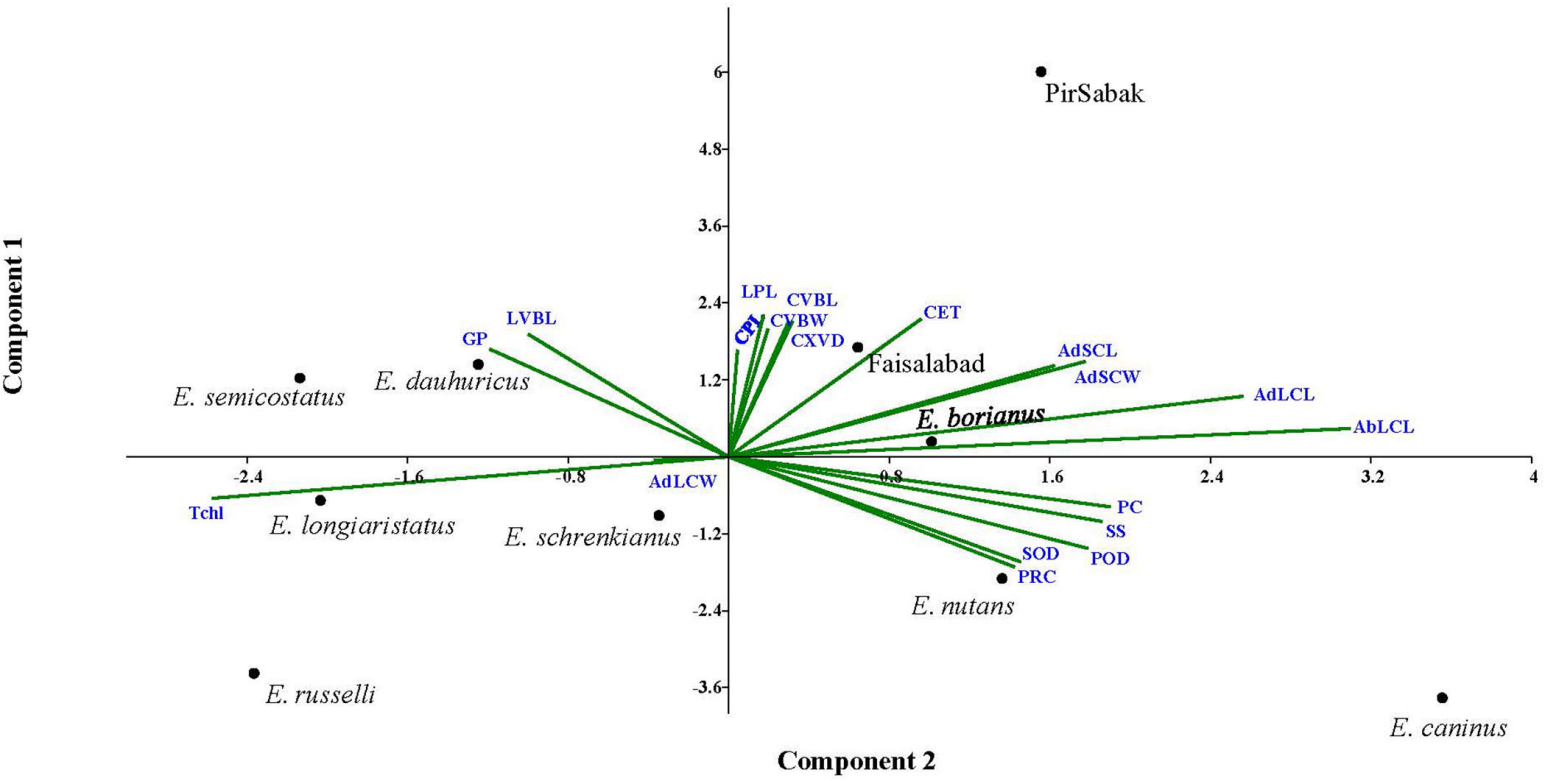
Principal component analysis (PCA) of the studied traits in *Elymus species* and wheat cultivars. Trait vectors exhibiting angles ≤ 90° have a positive relationship, while those with angles depict negative associations ≥ 90°. PCA revealed up to 62.8% of the variability being explained by the first two PCs.

In search of finding the appropriate grouping of the studied traits in response to progressive water stress among *Elymus* species and wheat cultivars, principal component analysis (PCA) was carried out through mean values (Figure 5, Supplementary material 8). It was predicted that trait vectors exhibiting angles ≤ 90° have a positive relationship, while those with angles depict negative associations ≥90°. Further, the length of the vector in the PCA explains the extent of variation explained by the corresponding trait. Current PCA revealed up to 62.8% variability being explained by the first two axis i.e., PC1 (eigenvalue = 8.12) and PC2 (eigenvalue = 3.82). Considering the first 2 PCs, we observed that mostly morpho-physiological, biochemical, and leaf micromorphological traits contributed to PC1, while the attributes with a major contribution to PC2 were mostly anatomical (culm and leaf). Regarding contribution to PC1, the observed order of the traits included POD (0.83), ABLCL (0.82), SOD (0.74), PC (0.57), ADLCL (0.56), SS (0.52), and PRC (0.36). Similarly, regarding contribution to PC2, the observed contributing traits were ADSCL (0.80), ADLCL (0.79), LPL (0.79), ADSCW (0.74), CET (0.72), CVBW (0.66), LVBL (0.65), CVBL (0.65) and CXVD (0.51). For PC3, CXVD (0.61), CET (0.57), CVBW (0.45), CPL (0.442), and CVBL (0.44) were noted for their prominent contributions, while for PC4, only ADLCL (0.73), GP (0.48), and POD (0.38) were noted for their prominent contributions.

## Discussions

Germination and development of seedlings under laboratory conditions are considered suitable for evaluating plant responses to abiotic stresses. In the current study, the GPs of all the studied *Elymus* species were reduced significantly. It is a general observation that seeds experiencing osmotic stress hinder their ability to start the germination process efficiently, which may be correlated to the associated change in the activities of enzymes (Wei et al., 2021). This, in turn, leads to shrinkage in cell walls, lowering of turgor-dependent activities, including root elongation and leaf expansion, and ultimately, affecting crop establishment. Our results support the findings of Wu et al. (2019), who reported that an increase in PEG concentration resulted in increased MGT and reduced GP in *Echinochloa crusgalli*. Our results support the findings of Zhang and Nan (2010), who observed reduced germination in *E. dahuricus* populations under different osmotic potentials.

The photosynthetic efficiency of a plant in terms of light-harvesting capability is generally predicted through the exploration of its chlorophyll content (Ali et al., 2014b; Ayub et al., 2021). Plants exhibiting higher chlorophyll content are considered an index of drought tolerance. Hence, selection based on these traits may prevent growth inhibition and a reduction in yield under water stress (Ahmadi et al., 2018). In the present study, a mild decreasing trend in all the studied *Elymus* species and wheat cultivars were recorded regarding chlorophyll content, which may be attributed to their different tolerance responses and enhanced antioxidant and osmoprotectant activities under moderate and severe water stresses, respectively. This degradation of the absorbing pigments may be regarded as an avoidance mechanism since the production of ROS is mainly associated with excess energy absorption within the photosynthetic apparatus (Bouchemal et al., 2017). Further, variable reduction rates in chlorophyll content are also suggestive of the existence of genetic diversity among the studied *Elymus* species. Reports of such mild decreases or little altered chlorophyll contents have been reported by Xu and Zhou (2007) in Leymus Chinensis and Pour-Aboughadareh et al. (2017) in *Aegilops*-*Triticum* accessions.

Accumulation of compatible solutes, including Dhn (dehydrins) and LEA (late embryogenesis abundant) proteins, under scarce water availability, is indicative of plant drought tolerance. However, certain other proteins decline under water deficit conditions, and the extent of decline or accumulation depends largely on the genetic constitution of different plant species (Ahmadi et al., 2018). Similarly, soluble sugars are part of plant hydrolysis processes, have a role in signaling and sensing systems, and play a crucial role in plant metabolism. Soluble sugars also have a distinct osmoprotectant role in maintaining turgor pressure and stabilizing cellular membranes. Hence, differences in the accumulation of soluble sugars are considered among the genetic factors affecting plant performance under water deficit conditions. In the current study, a slight decline in protein content and enhanced accumulation of soluble sugars were observed, which indicated the existence of drought adaptive mechanisms in the studied *Elymus* species. A similar decrease in protein concentration and enhanced accumulation of soluble sugars under water deficit conditions have previously been reported by Hessini et al. (2009) and Xu and Zhou (2007), respectively. However, our findings do not support the findings of Borrajo et al. (2018), who reported an increase in protein content with an increase in the severity of drought stress, which may be due to differences in experimental plant materials and conditions of drought stress imposition. Further, the increased accumulation of soluble sugars may be attributed to reduced utilization of assimilates during drought due to inhibition of invertase or sucrose synthase activities (Lv et al., 2020).

Osmotic adjustment is a key mechanism of plants under drought stress conditions to maintain water uptake and cell wall pressure, keep stomata open, and keep physiological and biochemical processes intact (Chaves et al., 2009; Cyriac et al., 2018). Improved proline accumulation under water deficit conditions is considered advantageous as osmoregulation and desiccation protectant and, generally, is recommended as criteria for selection tolerance against drought stress (Gazanchian et al., 2007; Hessini et al., 2009; Noein and Soleymani, 2022). Our findings suggest that higher proline content improves a plant's ability to tolerate osmotic stress. Further, relative increase rather than absolute value could be the appropriate indicator of stress tolerance. The present study also revealed enhanced proline content in moderate and severe water stress. The findings of Ahmadi et al. (2018), Martinez et al. (2018), and Borrajo et al. (2018) are in general agreement with the results obtained in the current research, which suggests that the studied *Elymus* species may be a potential source for this important trait for tolerance to water deficit environments.

As a plant suffers from a water deficit condition, it not only experiences disturbance in cell turgidity and reduction in growth, but it also suffers from oxidative stress due to the presence of ROS. Removing these ROS plants possesses antioxidant mechanisms (Zhang and Nan, 2007; Bouchemal et al., 2017; Suneja et al., 2017). Peroxidase (POD) and superoxide dismutase (SOD) are major enzymes that play a key role in the defense mechanism of plants against various stress conditions by scavenging H_2_O_2_ in chloroplasts (Gill and Tuteja, 2010). Increased antioxidants, including superoxide dismutase and peroxidase, have an important role in coping with the oxidative damage caused by free radicals. Thus, they are considered important indices for assessing plant redox status and can be used to select drought-tolerant plants (Alscher et al., 2002). In the current study, drought stress up-regulated the activity of the antioxidative defense system, and we observed variable but enhanced SOD and POD activities in the studied plant materials, which suggests that the varied ROS-scavenging ability of *Elymus* species is a contributor to its improved drought tolerance. Our results generally agree with those reported by Suneja et al. (2017) and Ahmadi et al. (2018), who reported enhanced antioxidant activities in moderate and severe water stress compared to control.

A PCA biplot analysis can be utilized to select traits that can be categorized into main groups and subgroups based on homogeneity and dissimilarity. In our result, three groups of traits were identified in the PCA biplot, considering both PC1 and PC2 simultaneously. The PRC, SOD, POD, SS, and PC were clustered in group I, while CPL, LPL, CXVD, CVBL, CVBW, and CET were in group II, and GP and LVBL were in group III. Traits in the same group may be interpreted to exhibit closely related physio-biochemical and anatomical relationships (Abdi and Williams, 2010). Other researchers have widely and effectively used PCA biplot analysis for screening stress tolerance of experimental plant materials (Nouraein et al., 2013; Ali et al., 2015; Ahmed et al., 2019).

Diverse mechanisms through which adaptations to water stress are induced may include changes in the anatomy of plant root, stem, and leaf tissues that monitor the transfer of deleterious effects of elevated CO_2_ concentration and water deficit conditions across the cells (Engloner et al., 2003; Boughalleb et al., 2014). There was a decreasing trend in the studied culm anatomical attributes with increasing water deficit. For instance, phloem length and width, epidermal and ground tissue thickness, xylem vessel diameter, and vascular bundle length and width all exhibited progressive reduction with an increase in the severity of the water stress. These adaptive anatomical traits may be used to identify tolerant *Elymus* species in water-stress environments. Through current genetic engineering and molecular methods, they can be targeted for integration into susceptible kinds of wheat (Nassar et al., 2020; Liu et al., 2021). Reduced xylem vessel diameter in *Elymus* species subjected to moderate water stress may be related to the maintenance of the water conductivity, which may contribute to reduced susceptibility to xylem embolism (Lovisolo and Schubert, 1998; Haworth et al., 2017; Dolezal et al., 2019).

Regarding phloem length and width, the observed decrease in the water deficit environment may also be assumed as an adaptation strategy because water stress resulted in reduced growth in comparison to controlled treatment. The decline in both leaf and culm xylem vessel diameter may also be regarded as advantageous as it may reduce cavitation of the xylem vessels, which further suggests that *Elymus* species can thrive well in drought-stress environments through different anatomical adaptations. A similar reducing trend in anatomic attributes under progressive water stress has also been previously reported by El-Afry (2012), Selim and El-Nady (2011), Arantes et al. (2020), and David et al. (2017).

To save water, leaf anatomical adaptation to progressive water stress is also considered important for selection against plant drought tolerance (Boughalleb et al., 2014). In the present study, moderate and severe water stress highly affected leaf anatomical traits in the studied Elymus species and wheat cultivars. Similarly, we observed that while confronting moderate to severe water stress, a significant decrease in xylem vessel diameter, leaf lower and upper epidermal thicknesses, vascular bundle length and width, mesophyll tissue thickness, and phloem length and width were observed among *Elymus* species. This declining trend in leaf anatomical traits may be regarded as an important structural response to progressive water stress, probably affecting the conductance of CO_2_ diffusion (Wang et al., 2011). Hence, reduced tissue size in *Elymus* culm and leaves may be interpreted as a tolerance mechanism for maintaining turgor even under a lesser amount of water. We noted that progression from moderate to severe water stress led to a decrease in bundle sheath dimensions and a reduction in the thickness of both the xylem and phloem, suggesting the ability of the leaf to maintain water transport even under unfavorable conditions. For some of the studied attributes, slight enhancement was observed under moderate stress, which declined under severe stress. This signifies that *Elymus* grass may have leaf trait plasticity in response to unfavorable environmental conditions. Reduced stomatal length and width in comparison to controlled grown plants were also observed in all the studied *Elymus* species and wheat cultivars. This may be regarded as a dehydration avoidance mechanism for sustaining proper water status and reducing water loss under water deficit conditions (Xu and Zhou, 2008; Thangthong et al., 2019). Our findings are in general agreement with those previously reported by Wang et al. (2011), Selim and El-Nady (2011), Petrov et al. (2018), Arantes et al. (2020), Chartzoulakis et al. (2002), and Bosabalidis and Kofidis (2002). David et al. (2017), El-Afry (2012), and Selim et al. (2019).

Improvement in cultivated kinds of wheat may/can be achieved through approaches like mutation breeding, genetic transformation, and hybridizing wild resources (Ilyas et al., 2015; Kazi et al., 2015). Owing to the exceptional stress tolerance and disease resistance potential of the *Elymus* genus, it is considered a valuable gene pool that can be used for the genetic enhancement of crops like wheat, rye, barley, and wheat (Yang et al., 2017; Xiong et al., 2021; Tan et al., 2022; Zhang et al., 2022). For instance, ESTs identified by Habora et al. (2012) may locate introgression of *Leymus* chromosomes having genes linked with stress tolerance in cultivated wheat, which in turn can assist marker-assisted breeding. Similarly, *E. dahuricus*, which is found mainly in temperate zones, has been reported to be potentially salt tolerant (Borrajo et al., 2018). Moreover, *E. elongatus* subspecies *ponticus* has been reported to possess high water use efficiency and hence is regarded as potentially drought tolerant (Gazanchian et al., 2007). *Elymus* wawawaiensis is a long-lived and drought-tolerant wheat grass. *Elymus wawawaiensis, E. Canadensis, E. nutans*, and *E. glaucus* are also considered novel germplasm, having promising flooding and drought tolerance potential (Jensen et al., 2012; Lee et al., 2015). *Elymus repens* has been reported to possess a high level of resistance to Fusarium head blight and has, therefore, been recommended as an important genetic resource for breeding FHB-wheat (Zeng et al., 2013). *Elymus semicostatus* has recently been reported to exhibit high water use efficiency while maintaining stable relative water content and high chlorophyll despite being exposed to an unfavorable environment, suggesting it is a reservoir of diverse, tolerant genes (Kumar et al., 2022). In the current study, while comparing the overall performances of the studied plant materials (Supplementary material 9), an arbitrary scoring was done by assigning a score of 3 to the top 33% of *Elymus* species for any specific trait, a score of 2 to the next 33%, and a score of 1 to the remaining species. Accordingly, the maximum score was recorded for *E. borianus* (i.e., 39 in MS, 37 in SS, and 42 in both combined stresses), followed by *E*. *russelli, E*. *caninus, E*. *longioristatus*, and *E*. *dauhuricus*. Interestingly, in terms of stress tolerance, these *Elymus species* outperformed the check wheat cultivar, Pirsabak 2005, which is a rainfed variety. Conclusively, *E. borianus*, which is endemic to Swat, Pakistan, along with *E*. *russelli, E*. *caninus, E*. *longioristatus*, and *E*. *dauhuricus*. have proved as wild wheat relatives harbor the untapped genetic diversity for a variety of morpho-physiological and anatomical attributes under progressive water stress conditions.

## Conclusions

We investigated responses of *Elymus* species and wheat cultivars under osmotic (germination traits) and progressive water stress (physio-biochemical and anatomical characteristics). Several structural, morpho-physiological, and biochemical responses observed during the current study may be interpreted to have assisted these species in tolerating progressive water stress conditions. In conclusion, *E. borianus*, which is endemic to Swat, Pakistan, along with *E*. *russelli, E*. *caninus, E*. *longioristatus*, and *E*. *dauhuricus*, have proved wild wheat relatives harboring the untapped genetic diversity for a variety of the studied traits under progressive water stress conditions. Further, exploration/characterization of these *Elymus* species, including ex situ conservation/domestication and molecular confirmation, is suggested for the successful use and introgression of desirable traits in future wheat breeding programs.

## Data availability statement

The original contributions presented in the study are included in the article/Supplementary material, further inquiries can be directed to the corresponding author/s.

## Author contributions

AK, AA, and ZU designed the research. AK conducted the research. PK, AA, IA, and MA analyzed the data. AA, AR, and HS checked the final content of the manuscript. All authors contributed to the article and approved the submitted version.

## Conflict of interest

The authors declare that the research was conducted in the absence of any commercial or financial relationships that could be construed as a potential conflict of interest. The reviewer JI declared a shared affiliation with the author AR to the handling editor at the time of the review.

## Publisher's note

All claims expressed in this article are solely those of the authors and do not necessarily represent those of their affiliated organizations, or those of the publisher, the editors and the reviewers. Any product that may be evaluated in this article, or claim that may be made by its manufacturer, is not guaranteed or endorsed by the publisher.
