# Supplementary material for: Exploiting the drought tolerance of wild Elymus species for bread wheat improvement
Source: Front Plant Sci. 2022 Oct 6;13:982844. doi: 10.3389/fpls.2022.982844 (PMC9583530; doi:10.3389/fpls.2022.982844)
Supplement: Supplementary file 1 [file Data_Sheet_1.docx]

**ESM 1:** Physical and chemical properties of soil utilized for conducting pot experiment

| Texture | Silt loam |
| --- | --- |
| PH | 6.4 |
| Organic matter | 1.59 % |
| Calcium carbonate | 2.4 % |
| Nitrogen | 0.57 % |
| Phosphorus | 441 ppm |
| Potassium | 95 ppm |
| Copper | 2.9 ppm |
| Iron | 33.6 ppm |
| Zinc | 2.2 % |
| Manganese | 11.2 ppm |

**ESM 2:** Analysis of variance, mean values and percentage changes in leaf epidermal anatomy traits in *Elymus species* and wheat cultivars.

| **Source of variation** | **DF** | **Mean squares** | | | | | | | |
| --- | --- | --- | --- | --- | --- | --- | --- | --- | --- |
|  |  | **AbLCL** | **AbLCW** | **AbSCL** | **AbSCW** | **AdLCL** | **AdLCW** | **AdSCL** | **AdSCW** |
| **Plant materials** | 9 | 71053^***^ | 217.01^***^ | 1928.46^***^ | 502.94^***^ | 115423^***^ | 191.38^***^ | 2396.75^***^ | 471.935^***^ |
| **Drought stress** | 2 | 101232^***^ | 43 9.20^***^ | 314.61^***^ | 94.46^***^ | 49898^***^ | 437.019^***^ | 322.35^***^ | 154.01^***^ |
| **Drought stress x Plant materials** | 18 | 2610^NS^ | 13.76^NS^ | 20.52^*^ | 4.40^NS^ | 2655^NS^ | 24.189^NS^ | 66.52^***^ | 9.142_NS_ |
| **Error** | 261 | 8783 | 18.55 | 11.75 | 5.71 | 6345 | 26.71 | 13.88 | 7.5 |
| **Mean control** |  | 343.04 | 27.75 | 43.11 | 21.89 | 301.68 | 28.28 | 44.29 | 22.36 |
| **Mean moderate water stress** |  | 301.30 | 25.10 | 45.30 | 20.56 | 273.73 | 25.99 | 44.76 | 21.20 |
| **Mean severe water stress** |  | 280.57 | 23.62 | 46.62 | 20.00 | 257.52 | 24.11 | 47.60 | 19.88 |
| **%age change due to moderate stress** |  | 12.17 | 9.57 | -5.08 | 6.07 | 9.26 | 8.11 | -1.07 | 5.17 |
| **%age change due to severe Stress** |  | 18.21 | 14.90 | -8.15 | 8.63 | 14.64 | 14.76 | -7.49 | 11.09 |
| ***Where,* AbLCL,** abaxial surface long cells length; **AbLCW,** Abaxial surface long cells width; **AbSCL,** abaxial surface stomatal complex length; **AbSCW,** Abaxial surface stomatal complex width; **AdLCL,** Adaxial surface long cells length; **AdLCW,** Adaxial surface long cells width; **AdSCL,** Adaxial surface stomatal complex length; **AdSCW,** Adaxial surface stomatal complex width. | | | | | | | | | |

**ESM 3:** Analysis of variance, mean values and percentage change in culm anatomical traits in *Elymus species* and wheat cultivars.

|  |  | **Mean squares** | | | | | | |
| --- | --- | --- | --- | --- | --- | --- | --- | --- |
| **Source of variation** | **DF** | **CET** | **CXVD** | **CPL** | **CPW** | **CVBL** | **CVBW** | **CGT** |
| **Plant materials** | 9 | 26.29^***^ | 157.38^***^ | 405.99^***^ | 602.23^***^ | 6811.59^***^ | 5351.69^***^ | 148955^***^ |
| **Drought Stress** | 2 | 80.13^***^ | 78.19^***^ | 98.37^***^ | 90.83^***^ | 298.67^***^ | 121.38^NS^ | 706^*^ |
| **Drought Stress x Plant materials** | 18 | 0.718^NS^ | 0.97^NS^ | 1.25^NS^ | 1.72^NS^ | 12.92^NS^ | 2.37^NS^ | 143^NS^ |
| **Error** | 261 | 2.47 | 3.80 | 25.85 | 6.79 | 86.45 | 63.19 | 214.00 |
| **Mean Control** |  | 9.75 | 10.72 | 27.41 | 14.77 | 57.18 | 53.10 | 95.93 |
| **Mean Moderate Drought Stress** |  | 8.57 | 9.87 | 26.21 | 13.90 | 55.41 | 51.71 | 93.66 |
| **Mean Severe Drought Stress** |  | 7.99 | 8.95 | 25.44 | 12.86 | 53.72 | 50.93 | 90.63 |
| **%age Change due to Moderate Stress** |  | 12.10 | 7.92 | 4.36 | 5.88 | 3.09 | 2.63 | 2.36 |
| **%age Change due to Severe Stress** |  | 18.01 | 16.50 | 7.18 | 12.89 | 6.04 | 4.09 | 5.52 |
| ***Where,* CET,** culm epidermal thickness; **CXVD,** culm xylem vessel diameter; **CPL,** culm phloem length; **CPW,** culm phloem width, **CVBL,** culm vascular bundle length; **CVBW,** culm vascular bundle width; **CGT,** culm ground tissue thickness | | | | | | | | |

**ESM 4:** Analysis of variance, mean values and percentage changes in leaf anatomical traits in *Elymus species* and wheat cultivars.

|  |  | **Mean square** | | | | | | | |
| --- | --- | --- | --- | --- | --- | --- | --- | --- | --- |
| **Source of variation** | **DF** | **LUET** | **LLET** | **LXVD** | **LPL** | **LPW** | **LVBL** | **LVBW** | **LMT** |
| **Plant materials** | 9 | 60.79^***^ | 40.56^***^ | 140.76^***^ | 1629.96^***^ | 150.50^***^ | 2522.79^***^ | 2228.07^***^ | 4084.8^***^ |
| **Drought Stress** | 2 | 96.46^***^ | 106.51^***^ | 160.11^***^ | 168.69^*^ | 97.65^***^ | 287.36^*^ | 526.5^**^ | 277.63^NS^ |
| **Drought Stress x Plant materials** | 18 | 3.03^NS^ | 1.62^NS^ | 7.47^**^ | 2.66^NS^ | 1.65^NS^ | 4.37^NS^ | 45.89^NS^ | 6.26^NS^ |
| **Error** | 261 | 4.65 | 4.024 | 3.42 | 38.13 | 4.521 | 93.29 | 95.67 | 192.43 |
| **Mean Control** |  | 12 | 12.048 | 11.714 | 28.426 | 14.573 | 60.73 | 58.305 | 72.899 |
| **Mean Moderate Drought Stress** |  | 10.819 | 10.705 | 10.341 | 26.895 | 13.505 | 58.651 | 56.345 | 71.27 |
| **Mean Severe Drought Stress** |  | 10.05 | 10.019 | 9.1863 | 25.843 | 12.599 | 57.372 | 53.732 | 69.567 |
| **%age Change due to Moderate Stress** |  | 9.84 | 11.15 | 11.72 | 5.39 | 7.33 | 3.42 | 3.36 | 2.23 |
| **%age Change due to Severe Stress** |  | 16.25 | 16.84 | 21.58 | 9.09 | 13.55 | 5.53 | 7.84 | 4.57 |
| ***Where,* LUET,** Leaf upper epidermis thickness; **LLET,**  Leaf lower epidermal thickness; **LXVD,**  Leaf xylem vessel diameter; **LPL,** Leaf phloem length; **LPW,** Leaf phloem width; **LVBL,** Leaf vascular bundle length; **LVBW,**  Leaf vascular bundle width; **LMT,** Leaf mesophyll tissue thickness. | | | | | | | | | |

**ESM 5:** Variability in various traits of leaf epidermal anatomy in *Elymus species* and wheat cultivars under drought stress.

|  | **AbLCL (µm)** | | | | | **AbLCW (µm)** | | | | | **AbSCL (µm)** | | | | | **AbSCW (µm)** | | | | |
| --- | --- | --- | --- | --- | --- | --- | --- | --- | --- | --- | --- | --- | --- | --- | --- | --- | --- | --- | --- | --- |
|  | **%age change**  **to control** | | | | | **%age change**  **to control** | | | | | **%age change**  **to control** | | | | | **%age change**  **to control** | | | | |
| **Plant materials** | **Control** | **MS** | **SS** | **MS** | **SS** | **Control** | **MS** | **SS** | **MS** | **SS** | **Control** | **MS** | **SS** | **MS** | **SS** | **Control** | **MS** | **SS** | **MS** | **SS** |
| ***E. longioristatus*** | 237.82 | 220.55 | 217.42 | 7.26 | 8.58 | 24.30 | 22.02 | 21.25 | 9.39 | 12.56 | 40.69 | 43.49 | 46.08 | -6.88 | -13.23 | 21.17 | 20.45 | 19.69 | 3.40 | 6.98 |
| ***E. dahuricus*** | 360.81 | 290.24 | 263.22 | 19.56 | 27.05 | 32.80 | 28.43 | 26.07 | 13.31 | 20.52 | 48.20 | 53.01 | 54.88 | -9.96 | -13.84 | 24.18 | 21.69 | 21.11 | 10.29 | 12.68 |
| ***E. schrenkianus*** | 319.41 | 289.84 | 272.97 | 9.26 | 14.54 | 22.94 | 21.78 | 21.08 | 5.07 | 8.14 | 39.30 | 42.37 | 43.93 | -7.80 | -11.78 | 22.10 | 21.07 | 20.83 | 4.64 | 5.74 |
| ***E. semicostatus*** | 333.29 | 272.24 | 268.41 | 18.32 | 19.47 | 27.16 | 23.83 | 22.44 | 12.25 | 17.37 | 43.28 | 45.22 | 45.68 | -4.48 | -5.54 | 19.53 | 18.19 | 17.76 | 6.85 | 9.09 |
| ***E. nutans*** | 324.16 | 302.05 | 291.73 | 6.82 | 10.00 | 25.58 | 23.24 | 22.88 | 9.14 | 10.55 | 45.35 | 46.38 | 47.14 | -2.26 | -3.93 | 18.96 | 18.12 | 17.65 | 4.45 | 6.96 |
| ***E. russelli*** | 282.42 | 251.21 | 226.75 | 11.05 | 19.71 | 26.14 | 23.78 | 23.02 | 9.05 | 11.95 | 24.02 | 24.73 | 26.38 | -2.94 | -9.79 | 12.93 | 12.89 | 12.09 | 0.31 | 6.51 |
| ***E. borianus*** | 384.24 | 346.26 | 307.00 | 9.89 | 20.10 | 34.03 | 29.76 | 26.21 | 12.54 | 22.98 | 50.26 | 50.84 | 49.69 | -1.15 | 1.13 | 21.82 | 20.00 | 19.74 | 8.35 | 9.55 |
| ***E. caninus*** | 416.77 | 384.99 | 374.91 | 7.63 | 10.04 | 25.55 | 24.29 | 24.07 | 4.95 | 5.80 | 38.78 | 44.14 | 47.27 | -13.84 | -21.91 | 21.25 | 21.48 | 20.98 | -1.10 | 1.28 |
| **Pirsabak-2005** | 393.91 | 350.62 | 328.41 | 10.99 | 16.63 | 30.71 | 26.92 | 23.59 | 12.33 | 23.20 | 51.72 | 52.43 | 52.70 | -1.38 | -1.90 | 26.54 | 24.13 | 24.07 | 9.09 | 9.31 |
| **Faisalabad-2008** | 377.56 | 305.01 | 254.91 | 19.22 | 32.48 | 28.33 | 26.93 | 25.58 | 4.92 | 9.71 | 49.49 | 50.39 | 52.48 | -1.83 | -6.05 | 30.45 | 27.59 | 26.10 | 9.39 | 14.28 |
|  | **AdLCL (µm)** | | | | | **AdLCW (µm)** | | | | | **AdSCL (µm)** | | | | | **AdSCW (µm)** | | | | |
|  | **%age change**  **to control** | | | | | **%age change**  **to control** | | | | | **%age change**  **to control** | | | | | **%age change**  **to control** | | | | |
| **Plant materials** | **Control** | **MS** | **SS** | **MS** | **SS** | **Control** | **MS** | **SS** | **MS** | **SS** | **Control** | **MS** | **SS** | **MS** | **SS** | **Control** | **MS** | **SS** | **MS** | **SS** |
| ***E. longioristatus*** | 247.52 | 229.11 | 235.82 | 7.44 | 4.73 | 29.42 | 27.34 | 26.99 | 7.05 | 8.25 | 42.25 | 44.76 | 45.70 | -5.93 | -8.16 | 20.79 | 20.78 | 19.20 | 0.07 | 7.65 |
| ***E. dahuricus*** | 204.14 | 195.04 | 185.82 | 4.46 | 8.97 | 22.57 | 21.60 | 21.34 | 4.30 | 5.47 | 44.48 | 47.86 | 60.51 | -7.61 | -36.04 | 26.90 | 22.26 | 23.50 | 17.24 | 12.64 |
| ***E. schrenkianus*** | 289.40 | 273.49 | 266.80 | 5.50 | 7.81 | 28.07 | 27.07 | 23.80 | 3.55 | 15.19 | 42.30 | 42.27 | 43.16 | 0.08 | -2.03 | 19.86 | 19.96 | 17.32 | -0.50 | 12.81 |
| ***E. semicostatus*** | 332.58 | 291.82 | 283.63 | 12.25 | 14.72 | 30.64 | 28.50 | 24.39 | 6.98 | 20.39 | 44.96 | 44.74 | 45.94 | 0.48 | -2.20 | 23.10 | 22.74 | 19.52 | 1.56 | 15.52 |
| ***E. nutans*** | 348.45 | 303.67 | 284.89 | 12.85 | 18.24 | 26.00 | 23.73 | 23.44 | 8.74 | 9.83 | 46.40 | 45.11 | 47.34 | 2.79 | -2.03 | 21.93 | 21.00 | 20.60 | 4.22 | 6.03 |
| ***E. russelli*** | 165.74 | 156.73 | 152.34 | 5.44 | 8.09 | 28.14 | 27.83 | 22.46 | 1.11 | 20.18 | 21.51 | 22.29 | 23.52 | -3.64 | -9.33 | 12.43 | 11.43 | 11.20 | 7.99 | 9.87 |
| ***E. borianus*** | 320.26 | 304.92 | 270.29 | 4.79 | 15.60 | 34.18 | 32.10 | 27.60 | 6.09 | 19.25 | 51.99 | 52.34 | 53.31 | -0.67 | -2.53 | 21.46 | 20.69 | 18.71 | 3.61 | 12.82 |
| ***E. caninus*** | 339.42 | 325.51 | 312.91 | 4.10 | 7.81 | 25.96 | 23.67 | 25.32 | 8.81 | 2.46 | 44.16 | 44.67 | 50.74 | -1.15 | -14.90 | 23.29 | 23.05 | 21.76 | 1.03 | 6.59 |
| **Pirsabak-2005** | 408.18 | 349.07 | 307.07 | 14.48 | 24.77 | 29.78 | 25.39 | 22.01 | 14.74 | 26.07 | 53.50 | 52.82 | 53.67 | 1.27 | -0.32 | 25.30 | 25.06 | 22.24 | 0.96 | 12.10 |
| **Faisalabad-2008** | 361.09 | 307.91 | 275.65 | 14.73 | 23.66 | 28.09 | 22.66 | 23.74 | 19.33 | 15.49 | 51.30 | 50.73 | 52.15 | 1.12 | -1.66 | 28.52 | 25.05 | 24.73 | 12.16 | 13.27 |
| ***Where*, AbLCL,** abaxial surface long cells length; **AbLCW,** Abaxial surface long cells width; **AbSCL,** abaxial surface stomatal complex length; **AbSCW,** Abaxial surface stomatal complex width; **AdLCL,** Adaxial surface long cells length; **AdLCW,** Adaxial surface long cells width; **AdSCL,** Adaxial surface stomatal complex length; **AdSCW,** Adaxial surface stomatal complex width. | | | | | | | | | | | | | | | | | | | | |

**ESM 6:** Variability in various traits of culm anatomy in *Elymus ssp.* and wheat cultivars under drought stress.

|  | **CXVD (mm^2^)** | | | | | **CPL (µm)** | | | | | **CPW (µm)** | | | | |
| --- | --- | --- | --- | --- | --- | --- | --- | --- | --- | --- | --- | --- | --- | --- | --- |
|  | **%age change**  **to control** | | | | | **%age change**  **to control** | | | | | **%age change**  **to control** | | | | |
| **Plant materials** | **Control** | **MS** | **SS** | **MS** | **SS** | **Control** | **MS** | **SS** | **MS** | **SS** | **Control** | **MS** | **SS** | **MS** | **SS** |
| ***E. longioristatus*** | 9.30 | 8.30 | 7.26 | 10.78 | 21.98 | 24.54 | 23.76 | 22.80 | 3.17 | 7.11 | 13.45 | 12.59 | 11.97 | 6.37 | 10.99 |
| ***E. dahuricus*** | 11.09 | 10.13 | 9.29 | 8.62 | 16.20 | 29.70 | 28.25 | 27.68 | 4.88 | 6.80 | 12.53 | 13.20 | 11.26 | -5.35 | 10.14 |
| ***E. schrenkianus*** | 9.14 | 8.27 | 7.63 | 9.50 | 16.51 | 26.45 | 25.84 | 24.35 | 2.32 | 7.94 | 13.28 | 12.95 | 11.56 | 2.51 | 13.00 |
| ***E. semicostatus*** | 10.22 | 9.50 | 8.49 | 7.07 | 16.95 | 27.68 | 26.11 | 25.05 | 5.67 | 9.48 | 12.82 | 11.41 | 10.90 | 11.04 | 15.00 |
| ***E. nutans*** | 9.69 | 8.65 | 7.99 | 10.67 | 17.46 | 27.90 | 26.92 | 26.43 | 3.51 | 5.28 | 12.25 | 11.03 | 10.20 | 9.95 | 16.68 |
| ***E. russelli*** | 11.23 | 10.27 | 9.14 | 8.56 | 18.55 | 27.33 | 26.40 | 25.96 | 3.41 | 5.01 | 16.00 | 14.32 | 13.63 | 10.50 | 14.80 |
| ***E. borianus*** | 10.09 | 9.05 | 8.53 | 10.29 | 15.47 | 25.18 | 24.60 | 24.07 | 2.33 | 4.41 | 12.65 | 11.32 | 10.48 | 10.54 | 17.17 |
| ***E. caninus*** | 7.82 | 7.37 | 6.96 | 5.77 | 10.97 | 23.66 | 22.69 | 21.73 | 4.09 | 8.14 | 10.34 | 9.19 | 8.19 | 11.10 | 20.74 |
| **Pirsabak-2005** | 16.79 | 16.10 | 13.87 | 4.09 | 17.39 | 36.82 | 35.06 | 34.28 | 4.78 | 6.90 | 25.59 | 24.67 | 24.28 | 3.62 | 5.13 |
| **Faisalabad-2008** | 11.85 | 11.08 | 10.36 | 6.50 | 12.56 | 24.79 | 22.47 | 22.02 | 9.38 | 11.20 | 18.77 | 18.32 | 16.17 | 2.37 | 13.84 |
|  | **CVBL (µm)** | | | | | **CVBW (µm)** | | | | | **CGT (µm)** | | | | |
|  | **%age change**  **to control** | | | | | **%age change**  **to control** | | | | | **%age change**  **to control** | | | | |
|  | **Control** | **MS** | **SS** | **MS** | **SS** | **Control** | **MS** | **SS** | **MS** | **SS** | **Control** | **MS** | **SS** | **MS** | **SS** |
| ***E. longioristatus*** | 51.52 | 49.86 | 48.52 | 3.23 | 5.82 | 49.44 | 47.23 | 47.00 | 4.47 | 4.93 | 88.20 | 87.55 | 85.30 | 0.73 | 3.29 |
| ***E. dahuricus*** | 52.68 | 50.91 | 49.68 | 3.36 | 5.68 | 48.84 | 47.68 | 47.04 | 2.38 | 3.68 | 82.02 | 80.94 | 80.05 | 1.32 | 2.41 |
| ***E. schrenkianus*** | 55.91 | 53.51 | 50.13 | 4.28 | 10.34 | 50.99 | 48.22 | 47.59 | 5.43 | 6.67 | 66.16 | 65.99 | 64.03 | 0.26 | 3.23 |
| ***E. semicostatus*** | 53.27 | 52.15 | 50.85 | 2.10 | 4.53 | 50.20 | 49.84 | 48.56 | 0.72 | 3.27 | 70.08 | 68.90 | 67.27 | 1.68 | 4.01 |
| ***E. nutans*** | 48.75 | 47.38 | 45.75 | 2.81 | 6.16 | 46.69 | 44.21 | 43.40 | 5.30 | 7.04 | 54.82 | 53.31 | 51.63 | 2.76 | 5.81 |
| ***E. russelli*** | 52.54 | 51.27 | 50.57 | 2.43 | 3.75 | 48.99 | 47.31 | 46.82 | 3.42 | 4.42 | 89.44 | 88.52 | 86.16 | 1.03 | 3.67 |
| ***E. borianus*** | 53.54 | 52.10 | 51.92 | 2.69 | 3.02 | 52.13 | 50.67 | 50.13 | 2.80 | 3.85 | 70.05 | 68.93 | 66.30 | 1.59 | 5.35 |
| ***E. caninus*** | 38.06 | 37.77 | 36.93 | 0.76 | 2.95 | 37.38 | 36.47 | 35.05 | 2.43 | 6.25 | 48.63 | 47.33 | 45.39 | 2.68 | 6.66 |
| **Pirsabak-2005** | 98.05 | 93.86 | 89.42 | 4.27 | 8.80 | 87.09 | 86.74 | 85.82 | 0.40 | 1.46 | 303.48 | 289.55 | 276.78 | 4.59 | 8.80 |
| **Faisalabad-2008** | 67.47 | 65.29 | 63.45 | 3.24 | 5.97 | 59.29 | 58.69 | 57.90 | 1.02 | 2.36 | 86.37 | 85.55 | 83.37 | 0.94 | 3.47 |

**ESM 7:** Variability in various straits of leaf anatomy in *Elymus species* and wheat cultivars under drought stress.

|  | **LPL (µm)** | | | | | **LPW (µm)** | | | | | **LXVD (mm^2^)** | | | | |
| --- | --- | --- | --- | --- | --- | --- | --- | --- | --- | --- | --- | --- | --- | --- | --- |
|  | **%age change**  **to control** | | | | | **%age change**  **to control** | | | | | **%age change**  **to control** | | | | |
| **Plant materials** | **Control** | **MS** | **SS** | **MS** | **SS** | **Control** | **MS** | **SS** | **MS** | **SS** | **Control** | **MS** | **SS** | **MS** | **SS** |
| ***E. longioristatus*** | 25.04 | 23.19 | 22.01 | 7.38 | 12.09 | 14.03 | 12.55 | 11.75 | 10.56 | 16.23 | 9.14 | 8.22 | 7.94 | 10.07 | 13.15 |
| ***E. dahuricus*** | 29.82 | 28.70 | 28.24 | 3.76 | 5.32 | 16.16 | 15.44 | 15.10 | 4.47 | 6.53 | 9.93 | 8.73 | 8.14 | 12.11 | 18.00 |
| ***E. schrenkianus*** | 31.19 | 29.71 | 29.30 | 4.74 | 6.05 | 14.44 | 13.40 | 12.62 | 7.21 | 12.58 | 11.22 | 10.86 | 9.24 | 3.21 | 17.65 |
| ***E. semicostatus*** | 32.49 | 31.16 | 30.04 | 4.12 | 7.54 | 16.37 | 15.93 | 15.64 | 2.74 | 4.51 | 11.69 | 10.79 | 10.12 | 7.68 | 13.45 |
| ***E. nutans*** | 22.89 | 21.58 | 20.91 | 5.71 | 8.66 | 13.77 | 13.01 | 11.93 | 5.52 | 13.37 | 9.96 | 8.30 | 7.88 | 16.62 | 20.86 |
| ***E. russelli*** | 19.28 | 18.83 | 16.69 | 2.34 | 13.43 | 11.12 | 10.82 | 9.08 | 2.69 | 18.31 | 10.66 | 10.13 | 9.08 | 4.99 | 14.83 |
| ***E. borianus*** | 29.32 | 28.37 | 27.80 | 3.26 | 5.20 | 14.47 | 12.62 | 12.01 | 12.74 | 16.96 | 13.50 | 10.27 | 9.30 | 23.91 | 31.07 |
| ***E. caninus*** | 20.92 | 18.48 | 17.62 | 11.63 | 15.77 | 11.36 | 10.28 | 8.52 | 9.54 | 25.06 | 8.22 | 7.83 | 7.41 | 4.67 | 9.82 |
| **Pirsabak-2005** | 45.11 | 43.54 | 42.02 | 3.48 | 6.85 | 17.83 | 16.70 | 15.53 | 6.34 | 12.89 | 16.64 | 14.37 | 12.16 | 13.63 | 26.91 |
| **Faisalabad-2008** | 28.20 | 25.39 | 23.81 | 9.95 | 15.57 | 16.19 | 14.32 | 13.81 | 11.57 | 14.68 | 16.18 | 13.90 | 10.59 | 14.10 | 34.58 |
|  | **LMT (µm)** | | | | | **LVBL (µm)** | | | | | **LVBW (µm)** | | | | |
|  | **%age change**  **to control** | | | | | **%age change**  **to control** | | | | | **%age change**  **to control** | | | | |
|  | **Control** | **MS** | **SS** | **MS** | **SS** | **Control** | **MS** | **SS** | **MS** | **SS** | **Control** | **MS** | **SS** | **MS** | **SS** |
| ***E. longioristatus*** | 79.41 | 75.69 | 73.54 | 4.68 | 7.40 | 56.50 | 54.15 | 52.45 | 4.16 | 7.18 | 58.33 | 56.34 | 54.11 | 3.41 | 7.23 |
| ***E. dahuricus*** | 84.12 | 82.61 | 81.02 | 1.79 | 3.68 | 68.33 | 65.63 | 65.16 | 3.95 | 4.64 | 65.27 | 64.47 | 63.65 | 1.22 | 2.48 |
| ***E. schrenkianus*** | 73.12 | 72.58 | 71.31 | 0.74 | 2.48 | 68.35 | 66.95 | 65.45 | 2.04 | 4.24 | 62.22 | 60.36 | 59.51 | 2.98 | 4.35 |
| ***E. semicostatus*** | 86.31 | 84.78 | 82.64 | 1.77 | 4.25 | 69.74 | 67.57 | 66.79 | 3.11 | 4.23 | 64.73 | 63.40 | 50.37 | 2.06 | 22.18 |
| ***E. nutans*** | 51.57 | 50.59 | 49.48 | 1.91 | 4.07 | 54.35 | 52.22 | 49.50 | 3.91 | 8.91 | 53.93 | 51.34 | 49.25 | 4.81 | 8.68 |
| ***E. russelli*** | 67.54 | 65.21 | 62.29 | 3.45 | 7.77 | 51.12 | 49.43 | 47.19 | 3.29 | 7.67 | 48.38 | 46.21 | 44.00 | 4.48 | 9.06 |
| ***E. borianus*** | 78.55 | 77.13 | 74.30 | 1.81 | 5.41 | 63.46 | 61.04 | 62.10 | 3.81 | 2.14 | 63.15 | 59.05 | 58.27 | 6.50 | 7.73 |
| ***E. caninus*** | 55.63 | 53.04 | 52.05 | 4.66 | 6.45 | 44.14 | 43.00 | 41.87 | 2.57 | 5.14 | 41.68 | 39.91 | 38.86 | 4.25 | 6.76 |
| **Pirsabak-2005** | 81.50 | 80.26 | 79.48 | 1.52 | 2.47 | 72.67 | 69.66 | 67.90 | 4.15 | 6.56 | 70.69 | 69.29 | 67.19 | 1.98 | 4.95 |
| **Faisalabad-2008** | 71.25 | 70.81 | 69.58 | 0.61 | 2.34 | 58.66 | 56.86 | 55.31 | 3.07 | 5.71 | 54.68 | 53.09 | 52.10 | 2.91 | 4.71 |

**ESM 8**: Principal component analysis of the studied attributes in *Elymus species* and wheat cultivars

|  | **PC1** | **PC2** | **PC3** | **PC4** |
| --- | --- | --- | --- | --- |
| **Eigenvalue** | 8.12 | 3.82 | 2.26 | 1.96 |
| **% Variance** | 42.74 | 20.08 | 11.88 | 10.31 |
| **Cumulative Variance (%)** | 42.74 | 62.82 | 74.70 | 85.01 |
|  | Eigenvector | | | |
| **GP** | -0.4014 | 0.3556 | 0.676 | 0.478 |
| **SS** | 0.519 | -0.307 | -0.024 | -0.532 |
| **PC** | 0.572 | 0.052 | -0.297 | 0.301 |
| **PRC** | 0.364 | -0.398 | -0.477 | -0.313 |
| **Tchl** | -0.348 | -0.405 | -0.112 | 0.373 |
| **POD** | 0.832 | -0.341 | -0.004 | 0.379 |
| **SOD** | 0.746 | -0.642 | 0.027 | -0.036 |
| **AbLCL** | 0.824 | 0.386 | 0.305 | -0.224 |
| **AdLCL** | 0.563 | 0.795 | -0.186 | 0.079 |
| **AdLCW** | 0.0852 | 0.0742 | -0.031 | 0.729 |
| **AdSCL** | 0.172 | 0.800 | 0.115 | -0.083 |
| **AdSCW** | 0.1501 | 0.738 | 0.077 | -0.202 |
| **CET** | 0.027 | 0.718 | 0.567 | -0.012 |
| **CXVD** | -0.165 | 0.513 | 0.608 | -0.142 |
| **CPL** | -0.187 | 0.420 | 0.442 | -0.329 |
| **CVBL** | -0.186 | 0.648 | 0.438 | -0.134 |
| **CVBW** | -0.170 | 0.655 | 0.448 | -0.103 |
| **LPL** | -0.196 | 0.786 | 0.360 | -0.189 |
| **LVBL** | -0.496 | 0.650 | 0.293 | -0.127 |

|  | **ESM 9** | | |  |  |  |  |  |  |  |  |  |  |  |  |  |  |  |  |  |
| --- | --- | --- | --- | --- | --- | --- | --- | --- | --- | --- | --- | --- | --- | --- | --- | --- | --- | --- | --- | --- |
|  | **Traits scoring based on comparison between control and moderate stress treatment** | | | | | | | | | | | | | | | | | | | |
| S.No. | ***Elymus Species* / Wheat cultivar** | **AbLCL** | **AdLCL** | **AdSCL** | **AdSCW** | **CET** | **CXVD** | **CPL** | **CVBL** | **CVBW** | **LPL** | **LVBL** | **GP** | **SS** | **PC** | **PRC** | **Tchl** | **POD** | **SOD** | **Total score** |
| 1 | ***E. longioristatus*** | 3 | 2 | 3 | 3 | 1 | 1 | 3 | 2 | 1 | 1 | 1 | 3 | 1 | 1 | 2 | 2 | 2 | 1 | 33 |
| 2 | ***E. dauhuricus*** | 1 | 3 | 3 | 1 | 1 | 2 | 1 | 1 | 2 | 2 | 1 | 2 | 1 | 3 | 3 | 3 | 2 | 1 | 33 |
| 3 | ***E. schrenkianus*** | 2 | 2 | 2 | 3 | 1 | 1 | 3 | 1 | 1 | 2 | 3 | 1 | 2 | 1 | 3 | 1 | 3 | 2 | 34 |
| 4 | ***E. semicostatus*** | 1 | 1 | 1 | 2 | 2 | 2 | 1 | 3 | 3 | 2 | 2 | 3 | 1 | 2 | 1 | 2 | 3 | 1 | 33 |
| 5 | ***E. nutans*** | 3 | 1 | 1 | 1 | 3 | 1 | 2 | 2 | 1 | 1 | 1 | 1 | 2 | 1 | 2 | 1 | 1 | 2 | 27 |
| 6 | ***E. russelli*** | 1 | 2 | 3 | 1 | 2 | 2 | 2 | 3 | 1 | 3 | 2 | 1 | 3 | 3 | 2 | 3 | 2 | 1 | 37 |
| 7 | ***E. borianus*** | 2 | 3 | 2 | 2 | 2 | 1 | 3 | 2 | 2 | 3 | 2 | 2 | 2 | 1 | 3 | 1 | 3 | 3 | 39 |
| 8 | ***E. caninus*** | 3 | 3 | 2 | 2 | 1 | 3 | 2 | 3 | 2 | 1 | 3 | 3 | 1 | 2 | 1 | 2 | 1 | 2 | 37 |
| 9 | **Pirsabak 2005** | 2 | 1 | 1 | 3 | 3 | 3 | 1 | 1 | 3 | 3 | 1 | 1 | 3 | 3 | 1 | 1 | 1 | 3 | 35 |
| 10 | **Faisalabad 2008** | 1 | 1 | 1 | 1 | 3 | 3 | 1 | 1 | 3 | 1 | 3 | 2 | 3 | 2 | 1 | 3 | 1 | 3 | 34 |
|  | **Traits scoring based on comparison between control and severe stress treatment** | | | | | | | | | | | | | | | | | | | |
| S.No. |  | **AbLCL** | **AdLCL** | **AdSCL** | **AdSCW** | **CET** | **CXVD** | **CPL** | **CVBL** | **CVBW** | **LPL** | **LVBL** | **GP** | **SS** | **PC** | **PRC** | **Tchl** | **POD** | **SOD** | **Total score** |
| 1 | ***E. longioristatus*** | 3 | 3 | 2 | 3 | 1 | 1 | 2 | 2 | 1 | 1 | 3 | 3 | 1 | 1 | 3 | 3 | 3 | 1 | 37 |
| 2 | ***E. dauhuricus*** | 1 | 2 | 3 | 2 | 1 | 2 | 2 | 2 | 2 | 3 | 2 | 3 | 1 | 2 | 3 | 3 | 1 | 1 | 36 |
| 3 | ***E. schrenkianus*** | 2 | 3 | 1 | 1 | 1 | 2 | 1 | 1 | 1 | 3 | 3 | 3 | 3 | 1 | 3 | 1 | 2 | 3 | 35 |
| 4 | ***E. semicostatus*** | 2 | 2 | 2 | 1 | 3 | 2 | 1 | 2 | 3 | 2 | 1 | 2 | 1 | 2 | 1 | 1 | 2 | 1 | 31 |
| 5 | ***E. nutans*** | 2 | 1 | 1 | 3 | 2 | 1 | 3 | 1 | 1 | 2 | 1 | 2 | 2 | 1 | 1 | 2 | 1 | 3 | 30 |
| 6 | ***E. russelli*** | 1 | 2 | 3 | 2 | 3 | 1 | 3 | 3 | 2 | 1 | 1 | 2 | 3 | 3 | 2 | 1 | 1 | 2 | 36 |
| 7 | ***E. borianus*** | 1 | 1 | 2 | 1 | 2 | 3 | 3 | 3 | 2 | 3 | 2 | 1 | 2 | 1 | 2 | 3 | 3 | 2 | 37 |
| 8 | ***E. caninus*** | 3 | 3 | 3 | 3 | 1 | 3 | 1 | 3 | 1 | 1 | 2 | 1 | 2 | 2 | 1 | 2 | 2 | 2 | 36 |
| 9 | **Pirsabak 2005** | 2 | 1 | 1 | 2 | 2 | 1 | 2 | 1 | 3 | 2 | 3 | 1 | 3 | 3 | 2 | 1 | 3 | 3 | 36 |
| 10 | **Faisalabad 2008** | 1 | 1 | 1 | 1 | 3 | 3 | 1 | 1 | 3 | 1 | 1 | 1 | 1 | 3 | 1 | 2 | 1 | 1 | 27 |
|  | **Traits scoring based on comparison between control treatment and combined (moderate and severe) stresses** | | | | | | | | | | | | | | | | | | | |
| **S.No.** |  | **AbLCL** | **AdLCL** | **AdSCL** | **AdSCW** | **CET** | **CXVD** | **CPL** | **CVBL** | **CVBW** | **LPL** | **LVBL** | **GP** | **SS** | **PC** | **PRC** | **Tchl** | **POD** | **SOD** | **Total score** |
| 1 | ***E. longioristatus*** | 3 | 3 | 3 | 3 | 1 | 1 | 2 | 1 | 1 | 1 | 1 | 2 | 2 | 1 | 2 | 3 | 2 | 1 | 33 |
| 2 | ***E. dauhuricus*** | 1 | 2 | 3 | 1 | 1 | 2 | 1 | 2 | 2 | 3 | 2 | 3 | 1 | 2 | 3 | 3 | 2 | 1 | 35 |
| 3 | ***E. schrenkianus*** | 2 | 3 | 2 | 2 | 1 | 1 | 2 | 1 | 1 | 2 | 3 | 1 | 3 | 1 | 3 | 1 | 2 | 3 | 34 |
| 4 | ***E. semicostatus*** | 1 | 1 | 1 | 1 | 3 | 2 | 1 | 2 | 3 | 2 | 3 | 3 | 1 | 2 | 1 | 1 | 3 | 1 | 32 |
| 5 | ***E. nutans*** | 2 | 1 | 1 | 3 | 2 | 1 | 3 | 2 | 1 | 2 | 1 | 1 | 2 | 1 | 1 | 2 | 1 | 2 | 29 |
| 6 | ***E. russelli*** | 1 | 2 | 2 | 1 | 3 | 1 | 3 | 3 | 2 | 1 | 1 | 1 | 3 | 3 | 2 | 1 | 1 | 1 | 32 |
| 7 | ***E. borianus*** | 2 | 2 | 2 | 2 | 2 | 2 | 3 | 3 | 2 | 3 | 3 | 2 | 2 | 1 | 3 | 3 | 3 | 2 | 42 |
| 8 | ***E. caninus*** | 3 | 3 | 3 | 3 | 1 | 3 | 1 | 3 | 1 | 1 | 2 | 3 | 1 | 3 | 1 | 2 | 1 | 2 | 37 |
| 9 | **Pirsabak 2005** | 2 | 1 | 1 | 2 | 2 | 3 | 2 | 1 | 3 | 3 | 1 | 2 | 3 | 3 | 2 | 1 | 3 | 3 | 38 |
| 10 | **Faisalabad 2008** | 1 | 1 | 1 | 1 | 3 | 3 | 1 | 1 | 3 | 1 | 2 | 1 | 1 | 2 | 1 | 2 | 1 | 3 | 29 |
